# Supplementary material for: Best practices for multi-ancestry, meta-analytic transcriptome-wide association studies: Lessons from the Global Biobank Meta-analysis Initiative
Source: Cell Genom. 2022 Oct 12;2(10):100180. doi: 10.1016/j.xgen.2022.100180 (PMC9631681; doi:10.1016/j.xgen.2022.100180)
Supplement: Document S1. Figures S1–S16 and Tables S1–S5 [file mmc1.pdf]

Cell Genomics, Volume *2*

Supplemental information

Best practices for multi-ancestry, meta-analytic transcriptome-wide association studies: Lessons from the Global Biobank Meta-analysis Initiative

Arjun Bhattacharya, Jibril B. Hirbo, Dan Zhou, Wei Zhou, Jie Zheng, Masahiro Kanai, the Global Biobank Meta-analysis Initiative, Bogdan Pasaniuc, Eric R. Gamazon, and Nancy J. Cox

SUPPLEMENTAL INFORMATION

Best practices of multi-ancestry, meta-analytic transcriptome-wide associations: lessons from the Global Biobank Meta-Initiative

SUPPLEMENTAL FIGURES

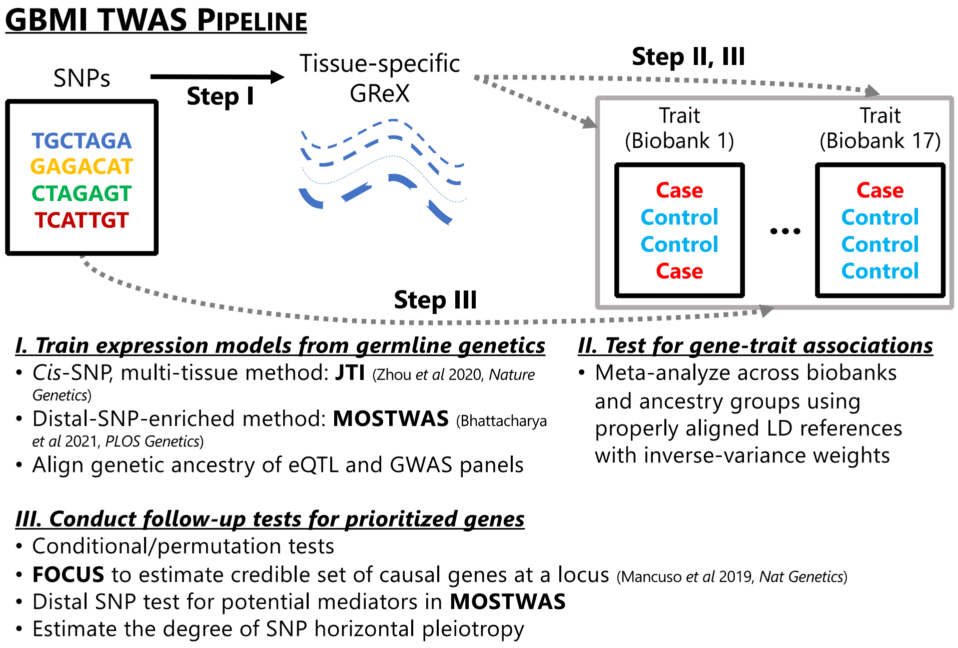

Figure S1: *An overview of the GBMI TWAS pipeline*. (Step 1) JTI and MOSTWAS for model training, (Step 2) inverse-variance weighted meta-analysis using per-biobank, per-ancestry group TWAS summary statistics, and (Step 3) various follow-up tests, including conditional or permutation tests, distal-SNPs added last test, probabilistic fine-mapping using FOCUS, and tests for SNP horizontal pleiotropy. Dotted lines represent associations that are tested in the TWAS pipeline, while the solid lines represent a link built through predictive modeling. Related to Figure 1.

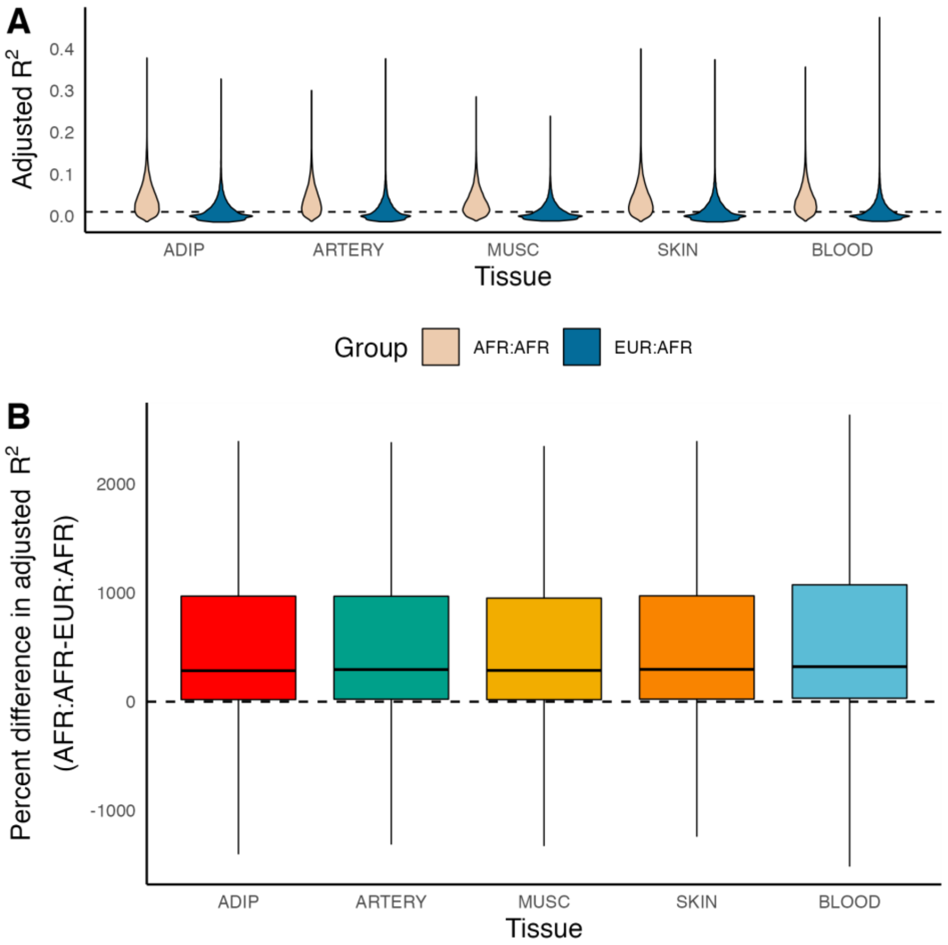

Figure S2: (A) Distribution of adjusted R^2 across 5 tissues using ancestry-aligned (AFR:AFR) models and ancestry-misaligned (EUR:AFR) models. (B) Boxplot of percent difference in adjusted R^2 using ancestry-aligned (AFR:AFR) models versus ancestry-misaligned (EUR:AFR) models. Related to Figure 2.

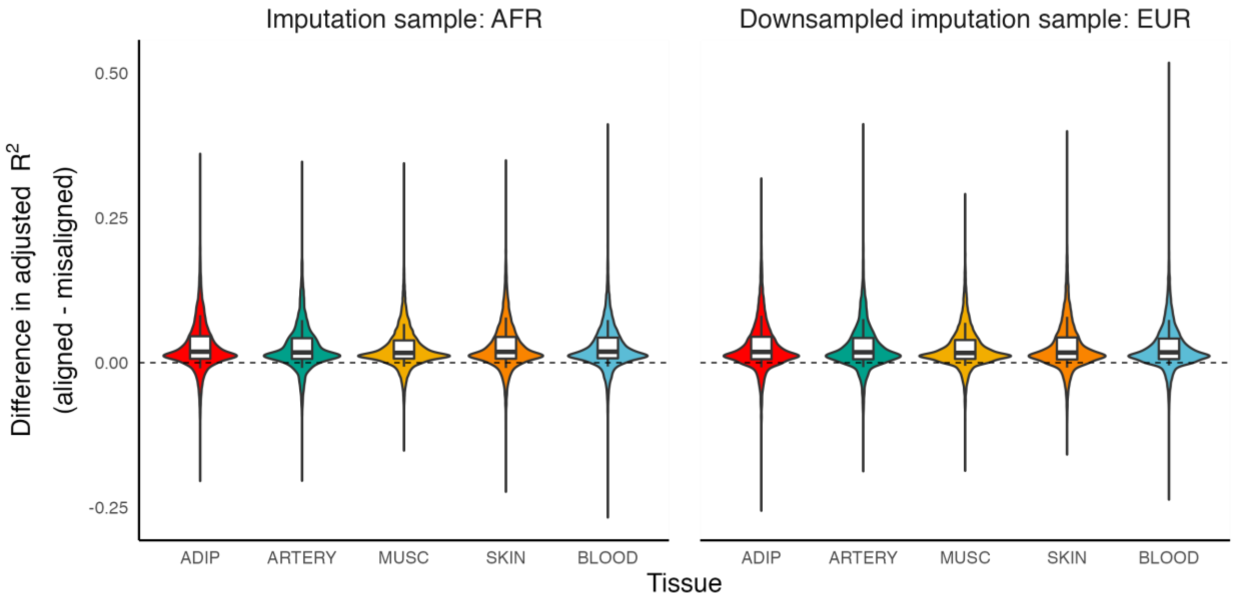

Figure S3: *Difference of predictive performance of expression models in aligned versus misaligned imputation samples across AFR (left) and EUR (right) ancestry in the imputation sample.* Here, we down-sample the EUR imputation sample to match the sample size of the AFR imputation sample. Related to Figure 2.

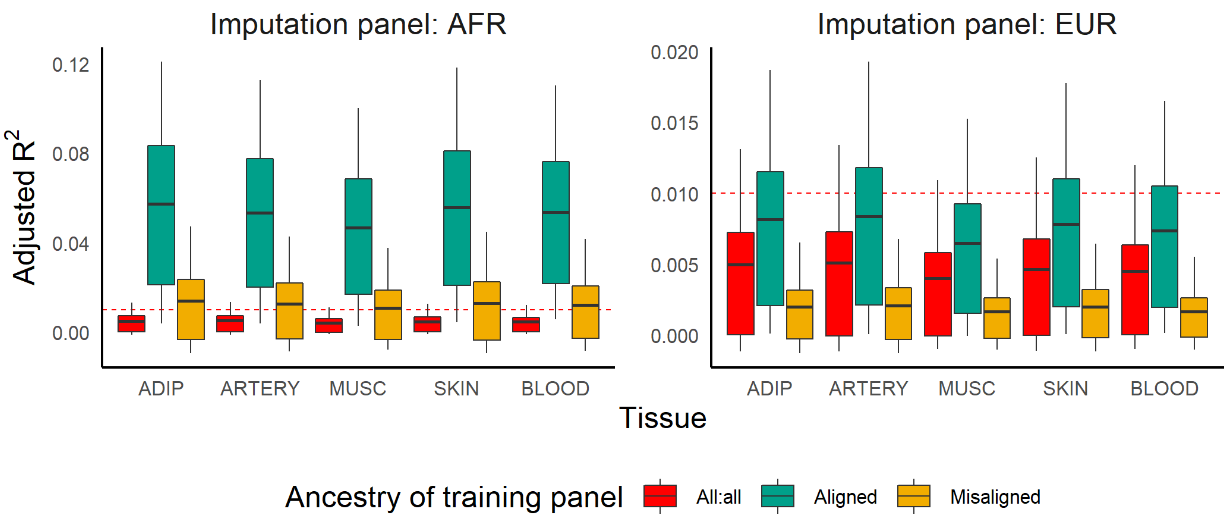

Figure S4: *Predictive performance of expression models in aligned and misaligned imputation samples*. Related to Figure 2.

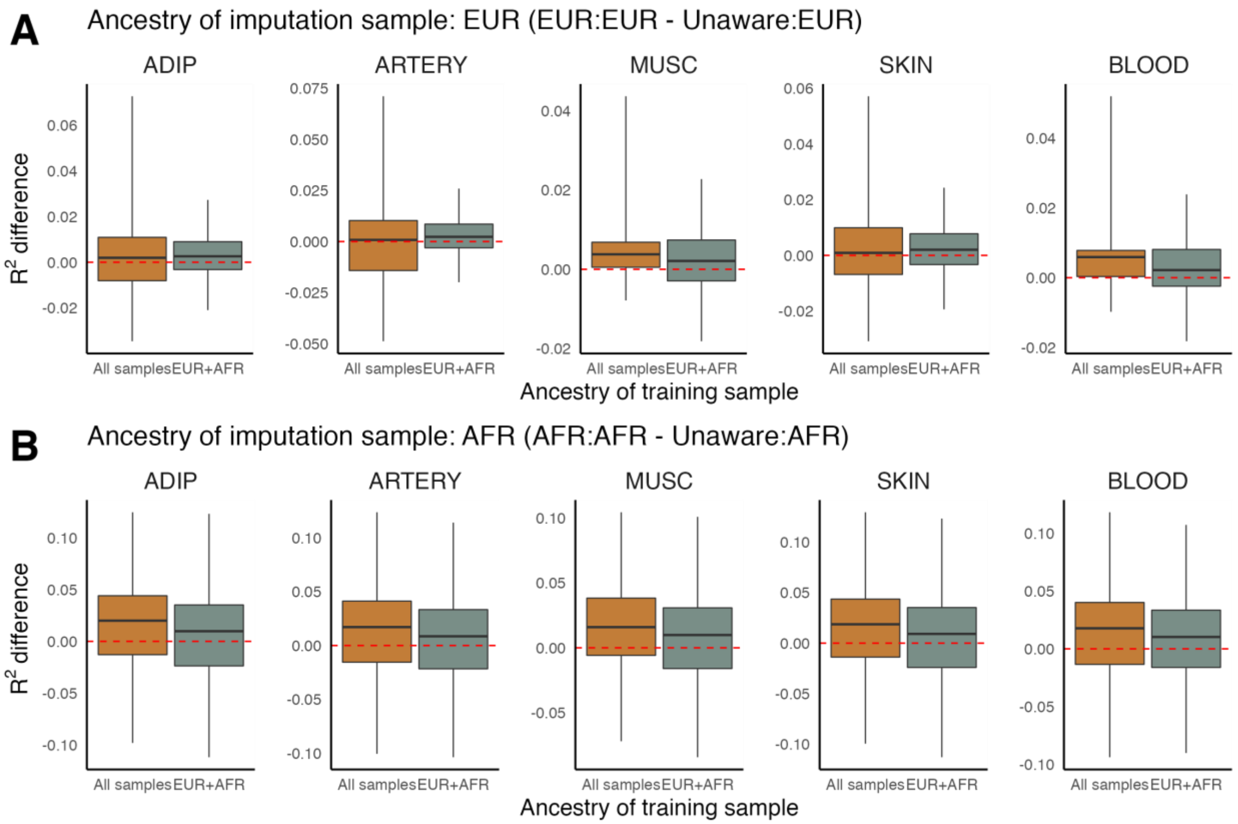

Figure S5: *Predictive performance of ancestry-unaware expression models compared to ancestry-specific models across 5 tissues.* Boxplot of difference in predictive performance in EUR **(A)** and AFR **(B)** samples between ancestry-aligned models and ancestry-unaware models. We consider (1) individuals of all ancestry in the training sample of the ancestry-unaware model (gold) or only EUR and AFR individuals in the training sample (grey). The red line indicates a difference of 0. Related to Figure 2.

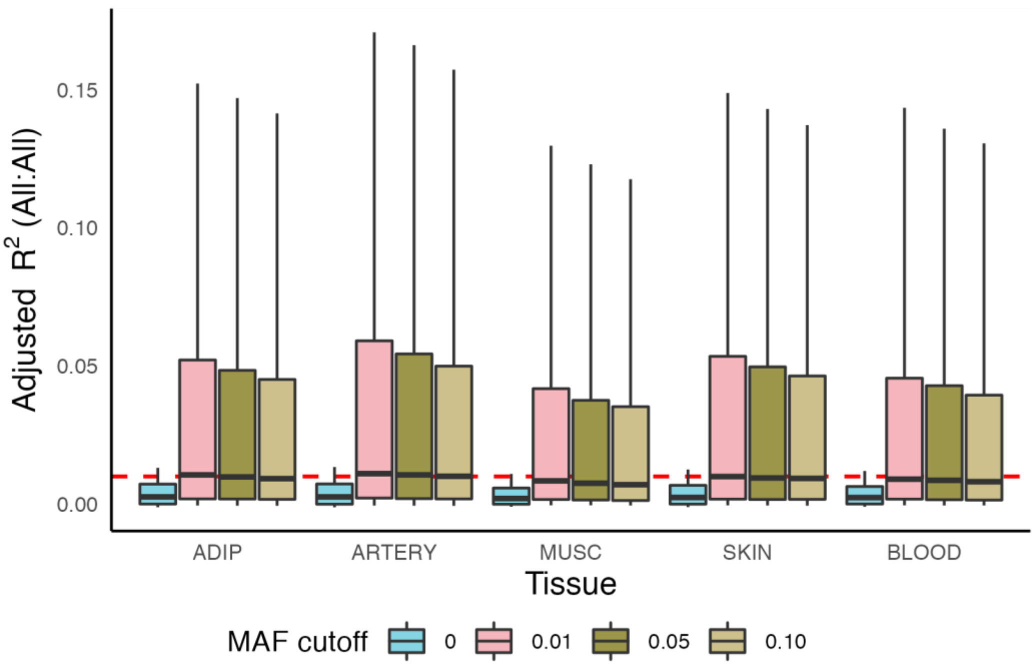

Figure S6: *Predictive performance of ancestry-unaware expression models across minor allele frequency thresholds*. Related to Figure 2.

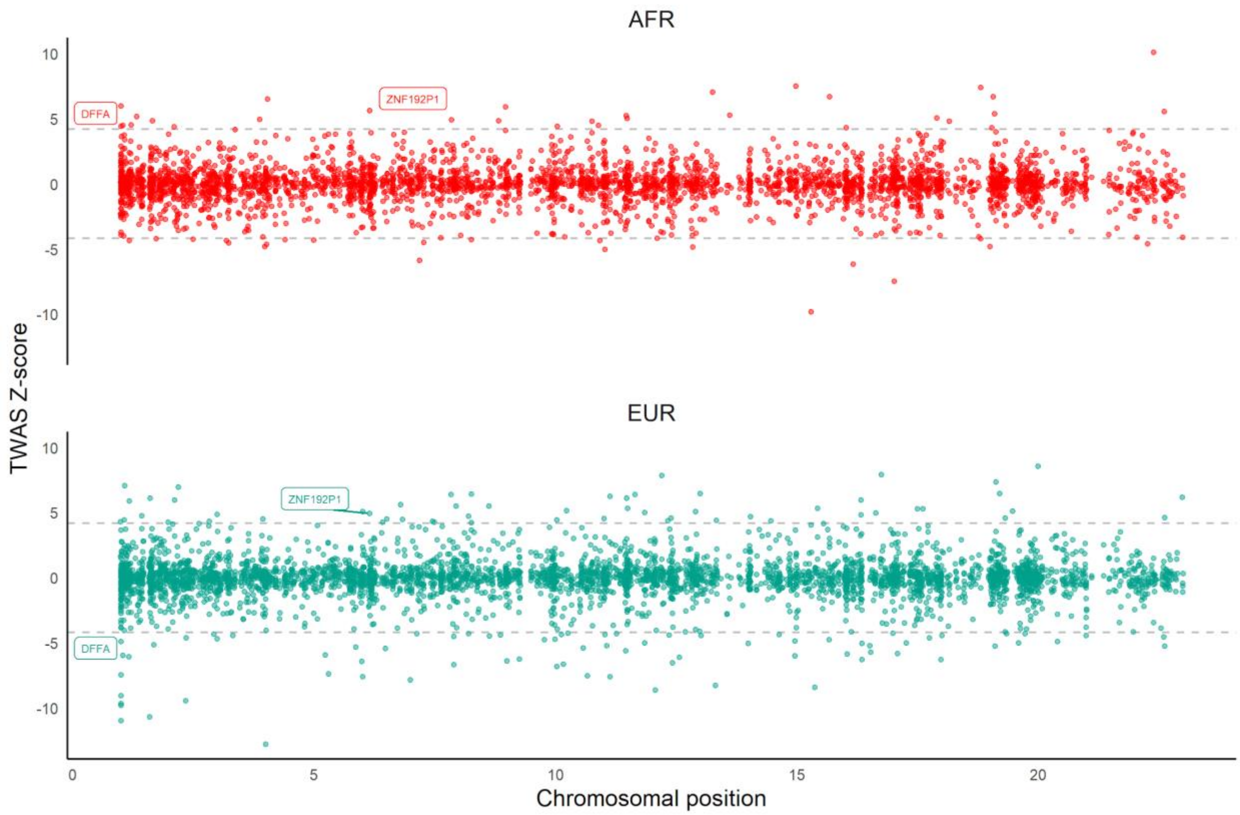

Figure S7: *TWAS Miami plots across AFR and EUR ancestry groups for asthma using whole blood gene expression models*. Related to Figure 3.

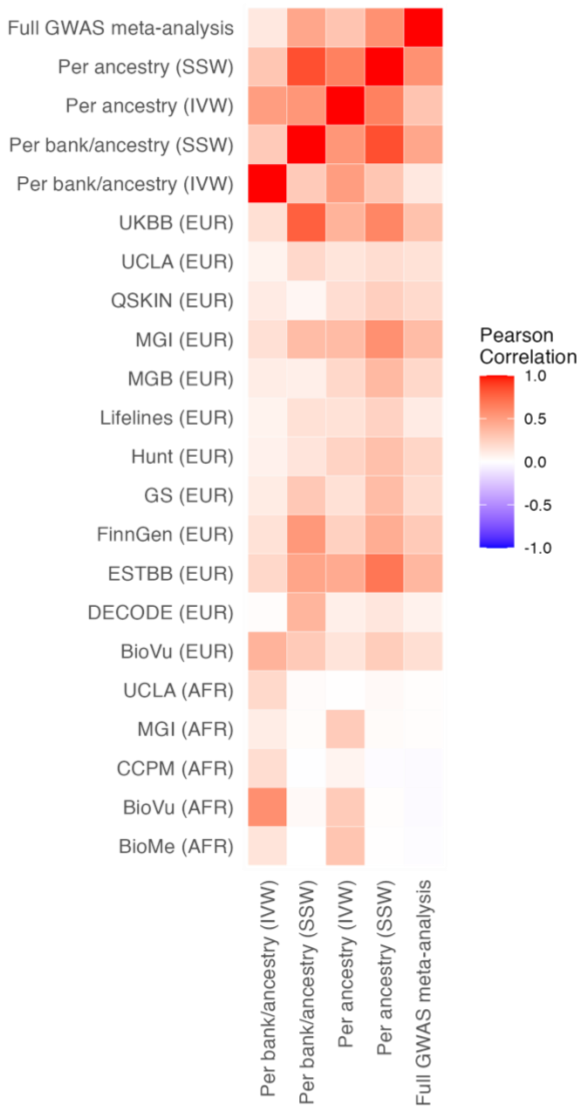

Figure S8: *Correlation of TWAS Z-scores across ancestry-specific, individual biobank GWAS cohorts and 5 meta-analytic strategies.* Related to Figure 3.

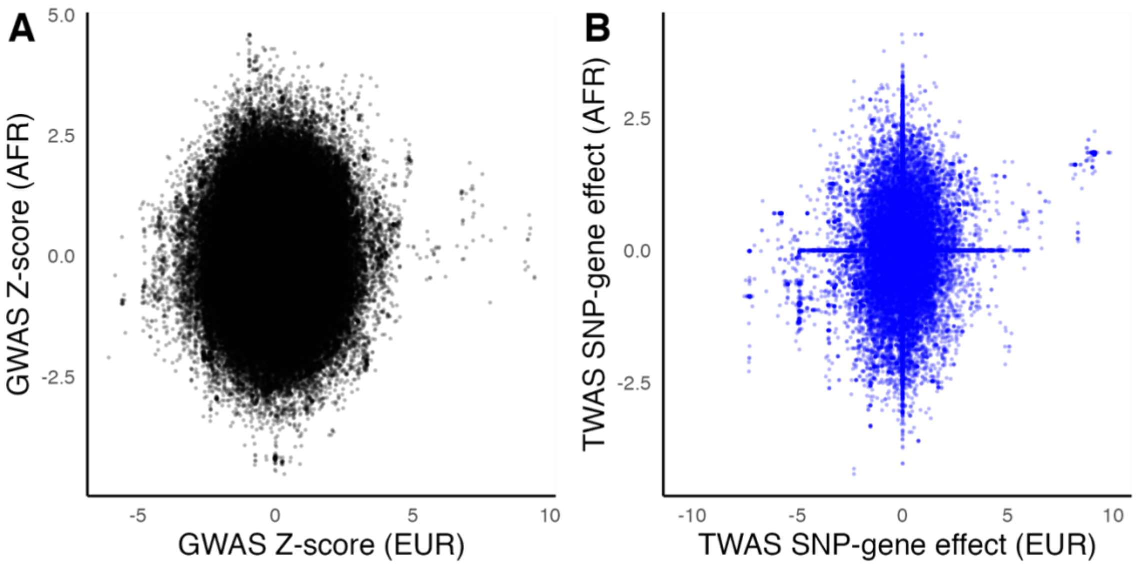

Figure S9: Correlation of meta-analyzed GWAS Z-scores (A) and TWAS SNP-gene effects (B) across EUR (X-axis) and AFR (Y-axis) ancestry groups. Related to Figure 3.

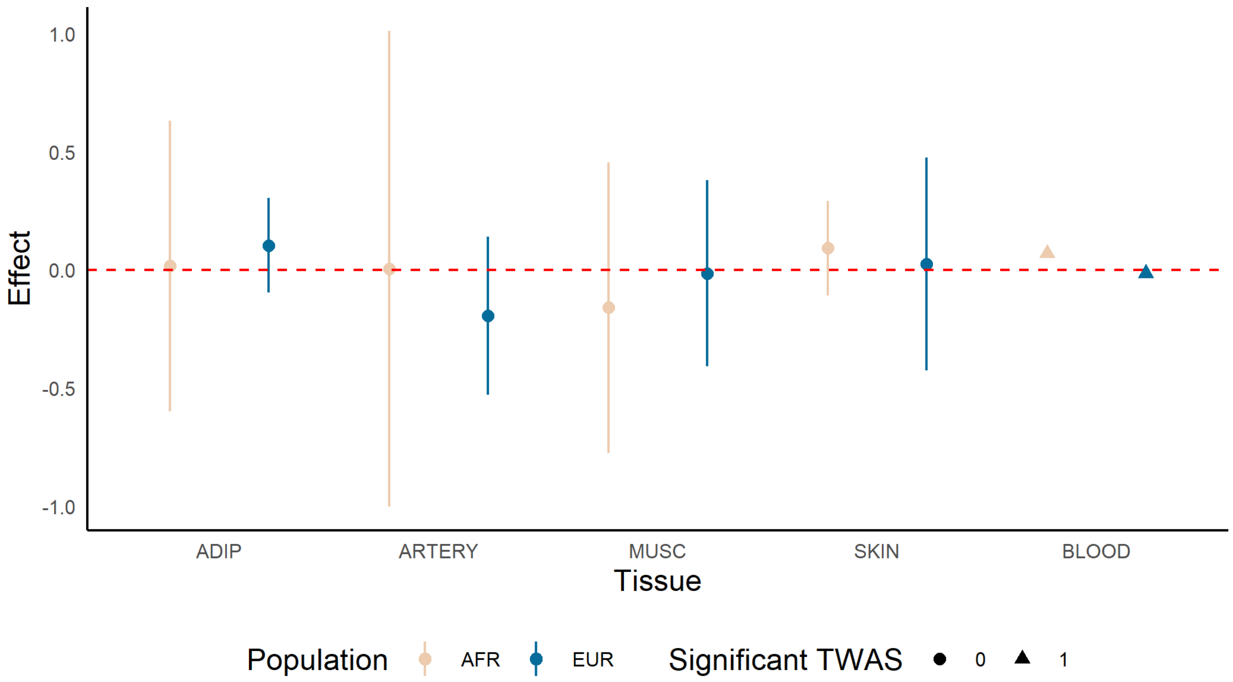

Figure S10: *TWAS associations across EUR and AFR ancestry groups for DFFA across 5 tissues.* The effect size is given with the point (triangle if association is transcriptome-wide significant) with a 95% confidence interval provided. Related to Figure 3.

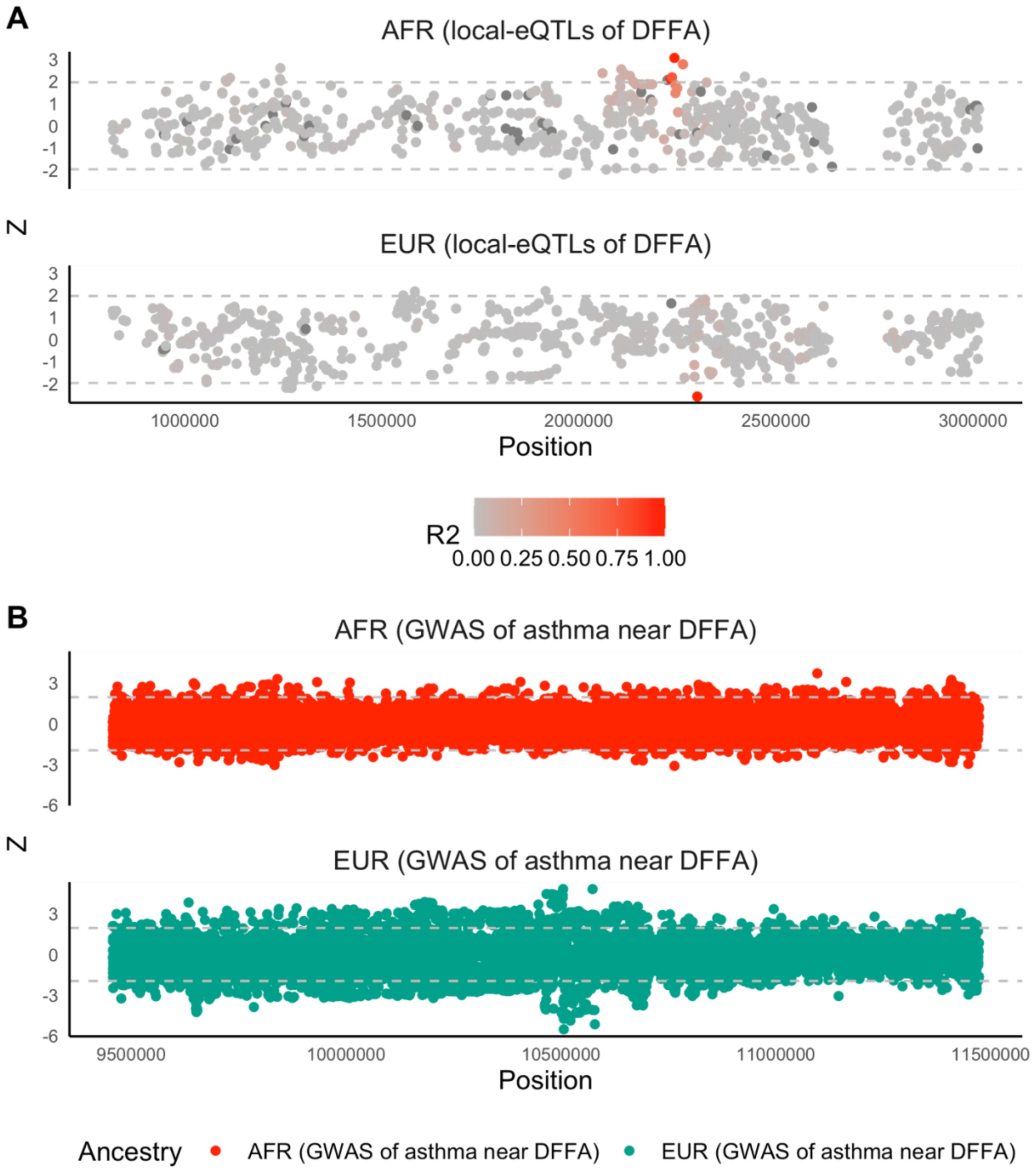

Figure S11: *Miami plots of DFFA local-eQTLs and GWAS signal for SNPs around DFFA.* In (A), color shows linkage disequilibrium R^2 to lead eQTL SNP. Grey line shows a nominal P-value cutoff of 0.05 (|Z| = 1.96). Related to Figure 3.

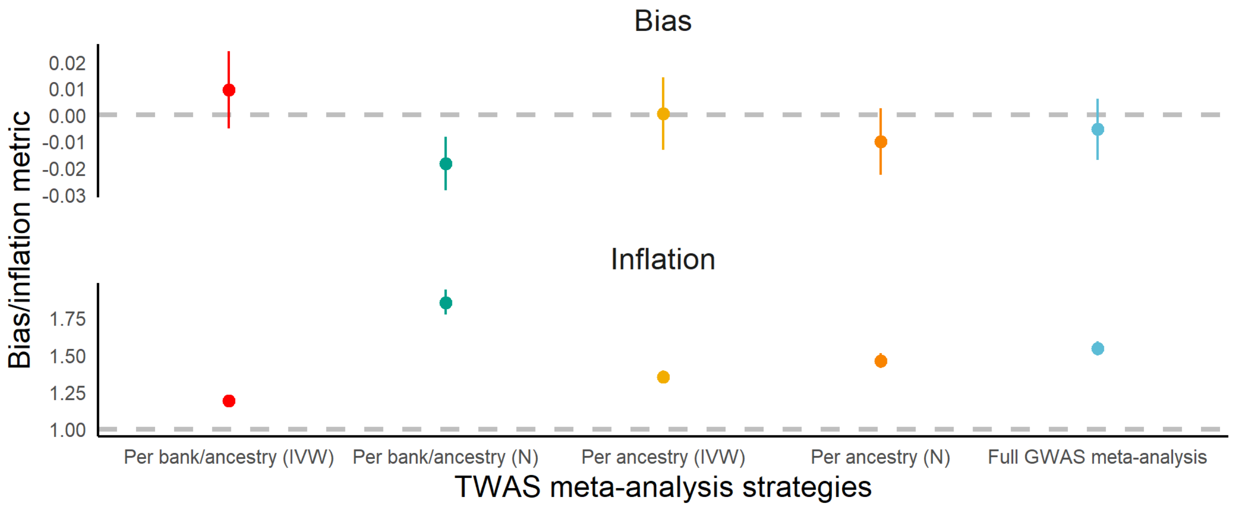

Figure S12: *Empirical Bayes estimates of bias and inflation in TWAS Z-scores across meta-analysis strategies.* Estimates of bias (top) and bottom (inflation) with one standard error width around the estimate are given across meta-analysis strategies. The dotted lines provide a reference for the null (0 for bias and 1 for inflation). Related to Figure 3.

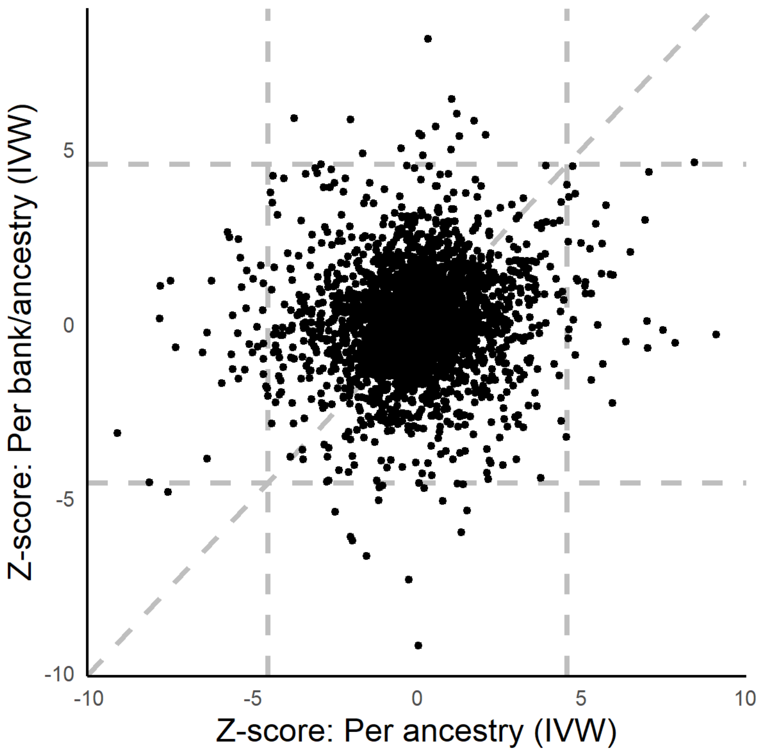

Figure S13: *Comparison of two IVW meta-analyzed Z-scores.* Vertical and horizontal dotted lines give a reference for the Bonferroni-corrected threshold for transcriptome-significance. A diagonal line is provided from reference. Related to Figure 3.

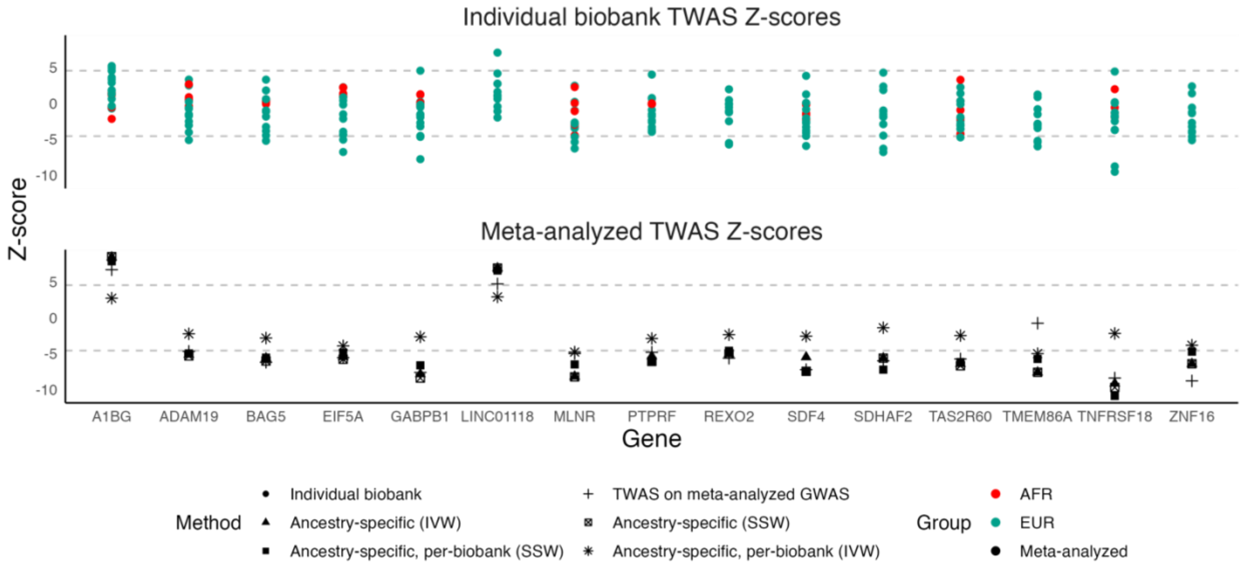

Figure S14: *Comparison of meta-analyzed Z-scores with individual biobank TWAS Z-scores.* Ancestry-specific TWAS Z-scores for individual biobanks are shown in the top panel, colored by ancestry. Meta-analyzed Z-scores are shown in the bottom panel with shapes reflecting the different strategies. Dotted lines provide a reference for transcriptome-wide significance. Related to Figure 3.

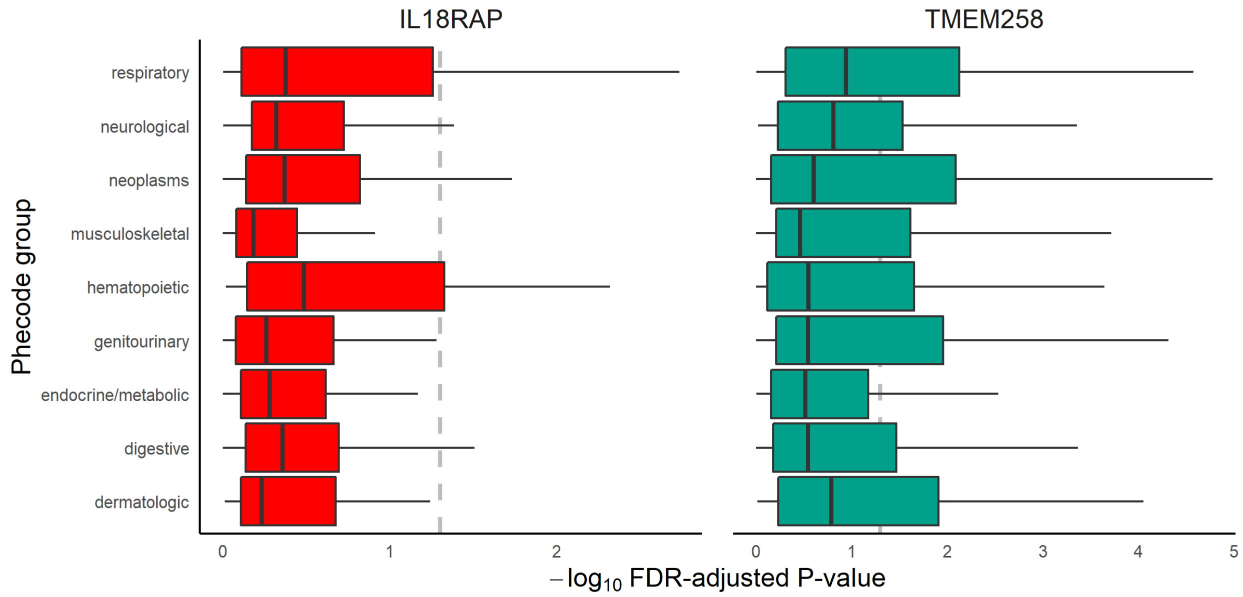

Figure S15: *UKBB GReX-PheWAS associations across 5 representative asthma-associated genes through European-only meta-analytic TWAS, grouped by phecode group.* The horizontal dotted line shows FDR-adjusted P = 0.05. Related to Figure 4.

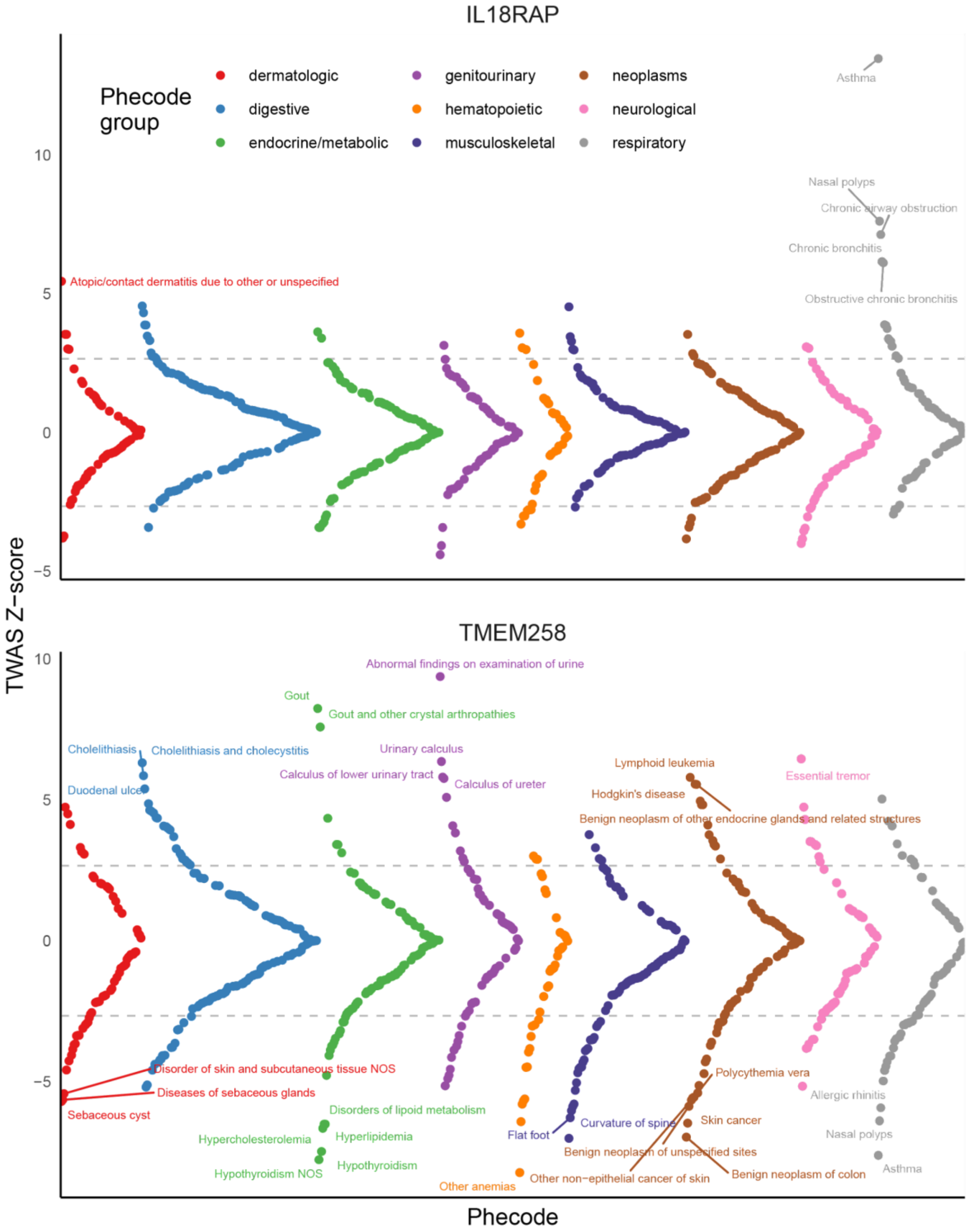

Figure S16: *Miami plots of UKBB GReX-PheWAS associations across 2 genes previously implicated through GWAS and detected in European-only meta-analytic TWAS in GBMI.* Related to Figure 4.

SUPPLEMENTAL TABLES

Table S1: Difference in adjusted R2 between models trained in aligned and misaligned ancestry samples as the ancestry of the imputation sample. Related to Figure 2.

	Imputation sample ancestry: down-sampled EUR					Imputation sample ancestry: AFR				
**Tissue**	**Mean**	**Median**	**25% Quantile**	**75% Quantile**	**Percent > 0**	**Mean**	**Median**	**25% Quantile**	**75% Quantile**	**Percent > 0**
**ADIP**	0.018	0.009	3.77E-04	3.50E-02	73.4	0.018	0.009	0.008	0.036	77.6
**ARTERY**	0.017	0.008	3.00E-04	3.30E-02	73.5	0.016	0.008	0.008	0.033	78.3
**MUSC**	0.015	0.007	5.27E-04	2.90E-02	74.1	0.015	0.008	0.010	0.036	77.4
**SKIN**	0.017	0.007	1.89E-04	3.40E-02	72.5	0.010	0.009	0.006	0.034	78.2
**BLOOD**	0.017	0.008	3.12E-04	3.20E-02	73.7	0.017	0.009	0.008	0.033	79.4

Table S2: R^2 of ancestry-specific models imputed into EUR imputation sample (training:imputation). Related to Figure 2.

	EUR:EUR				AFR:EUR			
**Tissue**	**Mean**	**Median**	**25% Quantile**	**75% Quantile**	**Mean**	**Median**	**25% Quantile**	**75% Quantile**
**ADIP**	0.008	0.006	2.02E-03	1.12E-02	0.002	0.001	-2.64E-04	3.19E-03
**ARTERY**	0.008	0.006	2.05E-03	1.15E-02	0.002	0.001	-3.15E-04	3.33E-03
**MUSC**	0.007	0.005	1.50E-03	9.00E-03	0.002	0.001	-2.29E-04	2.64E-03
**SKIN**	0.008	0.006	1.90E-03	1.10E-02	0.002	0.001	-1.85E-04	3.20E-03
**BLOOD**	0.007	0.005	1.92E-03	1.03E-02	0.002	0.001	-1.22E-04	2.64E-03

Table S3: R^2 of ancestry-specific models imputed into AFR imputation sample (training:imputation). Related to Figure 2.

	AFR:AFR				EUR:AFR			
**Tissue**	**Mean**	**Median**	**25% Quantile**	**75% Quantile**	**Mean**	**Median**	**25% Quantile**	**75% Quantile**
ADIP	0.043	0.035	0.015	0.062	0.014	0.006	-0.003	0.023
ARTERY	0.040	0.033	0.015	0.058	0.013	0.005	-0.003	0.022
MUSC	0.035	0.028	0.012	0.051	0.011	0.004	-0.003	0.019
SKIN	0.042	0.034	0.015	0.060	0.013	0.005	-0.004	0.023
BLOOD	0.040	0.034	0.016	0.057	0.012	0.005	-0.003	0.021

Table S4: Difference in R^2 between ancestry-specific and ancestry-unaware models across MAF. Related to Figure 2.

		AFR:AFR - All:AFR					EUR:EUR - All:EUR				
**Tissue**	**MAF**	**Mean**	**Median**	**25% Quantile**	**75% Quantile**	**Proportion > 0**	**Mean**	**Median**	**25% Quantile**	**75% Quantile**	**Proportion > 0**
**ADIP**	0	0.004	0.003	-0.003	0.009	0.634	0.028	0.024	-0.009	0.066	0.689
**ARTERY**	0	0.003	0.002	-0.003	0.009	0.620	0.026	0.022	-0.010	0.060	0.679
**MUSC**	0	0.004	0.002	-0.003	0.008	0.625	0.025	0.022	-0.014	0.061	0.664
**SKIN**	0	0.003	0.002	-0.003	0.008	0.622	0.023	0.020	-0.009	0.053	0.674
**BLOOD**	0	0.003	0.002	-0.003	0.008	0.635	0.028	0.027	-0.004	0.060	0.724
**ADIP**	0.01	-0.001	-0.001	-0.006	0.004	0.375	0.026	0.028	0.002	0.086	0.750
**ARTERY**	0.01	0.003	0.002	-0.003	0.009	0.632	0.025	0.023	-0.010	0.058	0.689
**MUSC**	0.01	0.003	0.002	-0.003	0.008	0.621	0.027	0.026	-0.010	0.062	0.692
**SKIN**	0.01	0.003	0.002	-0.002	0.007	0.623	0.022	0.021	-0.007	0.050	0.695
**BLOOD**	0.01	0.003	0.002	-0.003	0.007	0.620	0.030	0.029	-0.005	0.064	0.715
**ADIP**	0.05	0.019	0.002	-0.003	0.007	0.686	0.026	0.023	-0.008	0.061	0.692
**ARTERY**	0.05	0.004	0.003	-0.003	0.010	0.644	0.024	0.023	-0.009	0.057	0.688
**MUSC**	0.05	0.004	0.002	-0.003	0.008	0.610	0.026	0.025	-0.010	0.063	0.689
**SKIN**	0.05	0.003	0.002	-0.003	0.007	0.621	0.022	0.021	-0.010	0.053	0.688
**BLOOD**	0.05	0.003	0.002	-0.003	0.008	0.621	0.027	0.025	-0.005	0.060	0.719

Table S5: Cross-validation R^2 of ancestry-unaware models across MAF threshold. Related to Figure 2.

Tissue	MAF	Mean	Median	25% Quantile	75% Quantile
ADIP	0.00	0.005	0.003	8.56E-06	7.23E-03
ADIP	0.01	0.051	0.011	1.88E-03	5.21E-02
ADIP	0.05	0.049	0.010	1.83E-03	4.84E-02
ADIP	0.10	0.047	0.009	1.72E-03	4.51E-02
ARTERY	0.00	0.005	0.003	-4.12E-05	7.28E-03
ARTERY	0.01	0.056	0.011	2.17E-03	5.91E-02
ARTERY	0.05	0.054	0.011	1.97E-03	5.43E-02
ARTERY	0.10	0.052	0.010	1.86E-03	4.99E-02
BLOOD	0.00	0.005	0.002	2.20E-05	6.36E-03
BLOOD	0.01	0.047	0.009	1.79E-03	4.56E-02
BLOOD	0.05	0.045	0.009	1.57E-03	4.29E-02
BLOOD	0.10	0.043	0.008	1.44E-03	3.94E-02
MUSC	0.00	0.004	0.002	-5.80E-05	5.80E-03
MUSC	0.01	0.043	0.008	1.66E-03	4.17E-02
MUSC	0.05	0.041	0.008	1.48E-03	3.75E-02
MUSC	0.10	0.039	0.007	1.29E-03	3.53E-02
SKIN	0.00	0.005	0.002	-2.61E-05	6.79E-03
SKIN	0.01	0.051	0.010	1.79E-03	5.34E-02
SKIN	0.05	0.049	0.009	1.67E-03	4.97E-02
SKIN	0.10	0.047	0.009	1.71E-03	4.63E-02
