# Supplementary material for: Best practices for multi-ancestry, meta-analytic transcriptome-wide association studies: Lessons from the Global Biobank Meta-analysis Initiative
Source: Cell Genom. 2022 Oct 12;2(10):100180. doi: 10.1016/j.xgen.2022.100180 (PMC9631681; doi:10.1016/j.xgen.2022.100180)
Supplement: Document S3. Article plus supplemental information [file mmc4.pdf]

Cell Genomics

Short Article

Best practices for multi-ancestry, meta-analytic transcriptome-wide association studies: Lessons from the Global Biobank Meta-analysis Initiative

Graphical abstract

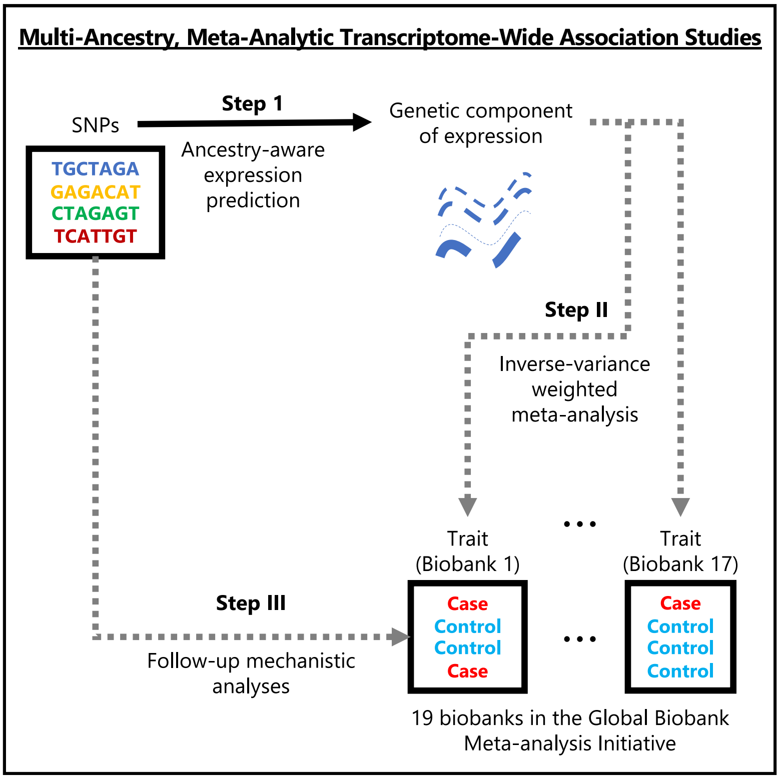

Highlights

- Expression prediction and gene-trait associations do not port across ancestry groups
- Recommend meta-analysis across both ancestry and study for least test statistic inflation
- Detail mechanistic tests to contextualize results from TWASs

Authors

Arjun Bhattacharya, Jibril B. Hirbo, Dan Zhou, ..., Bogdan Pasaniuc, Eric R. Gamazon, Nancy J. Cox

Correspondence

abtbhatt@ucla.edu

In brief

Bhattacharya and Hirbo et al. describe a framework to identify gene-level genetic associations with complex traits through meta-analysis of multi-ancestry datasets from biobanks using transcriptome-wide association studies (TWASs). They demonstrate pitfalls in TWASs and review open methodological challenges, including lack of portability of expression prediction and tissue specificity of results.

Bhattacharya et al., 2022, Cell Genomics *2*, 100180
October 12, 2022 © 2022 The Authors.
https://doi.org/10.1016/j.xgen.2022.100180

CellPress

Cell Genomics

CellPress
OPEN ACCESS

Short Article

Best practices for multi-ancestry, meta-analytic transcriptome-wide association studies: Lessons from the Global Biobank Meta-analysis Initiative

Arjun Bhattacharya,[1,2,14,15,*] Jibril B. Hirbo,[3,4,14] Dan Zhou,[3,4] Wei Zhou,[5,6,7] Jie Zheng,[8] Masahiro Kanai,[5,6,7,9,10] the Global Biobank Meta-analysis Initiative, Bogdan Pasaniuc,[1,11,12,14] Eric R. Gamazon,[3,4,13,14] and Nancy J. Cox[3,4,14]

[1]Department of Pathology and Laboratory Medicine, David Geffen School of Medicine, University of California, Los Angeles, Los Angeles, CA, USA

[2]Institute of Quantitative and Computational Biosciences, David Geffen School of Medicine, University of California, Los Angeles, Los Angeles, CA, USA

[3]Department of Medicine, Division of Genetic Medicine, Vanderbilt University School of Medicine, Nashville, TN, USA

[4]Vanderbilt Genetics Institute, Vanderbilt University Medical Center, Nashville, TN, USA

[5]Analytic and Translational Genetics Unit, Massachusetts General Hospital, Boston, MA, USA

[6]Program in Medical and Population Genetics, Broad Institute of Harvard and MIT, Cambridge, MA, USA

[7]Stanley Center for Psychiatric Research, Broad Institute of Harvard and MIT, Cambridge, MA, USA

[8]MRC Integrative Epidemiology Unit (IEU), Bristol Medical School, University of Bristol, Oakfield House, Oakfield Grove, Bristol BS8 2BN, UK

[9]Department of Biomedical Informatics, Harvard Medical School, Boston, MA, USA

[10]Department of Statistical Genetics, Osaka University Graduate School of Medicine, Suita 565-0871, Japan

[11]Department of Human Genetics, David Geffen School of Medicine, University of California, Los Angeles, Los Angeles, CA, USA

[12]Department of Computational Medicine, David Geffen School of Medicine, University of California, Los Angeles, Los Angeles, CA, USA

[13]MRC Epidemiology Unit, University of Cambridge, Cambridge, UK

[14]These authors contributed equally

[15]Lead contact

*Correspondence: abtbhatt@ucla.edu

https://doi.org/10.1016/j.xgen.2022.100180

SUMMARY

The Global Biobank Meta-analysis Initiative (GBMI), through its diversity, provides a valuable opportunity to study population-wide and ancestry-specific genetic associations. However, with multiple ascertainment strategies and multi-ancestry study populations across biobanks, GBMI presents unique challenges in implementing statistical genetics methods. Transcriptome-wide association studies (TWASs) boost detection power for and provide biological context to genetic associations by integrating genetic variant-to-trait associations from genome-wide association studies (GWASs) with predictive models of gene expression. TWASs present unique challenges beyond GWASs, especially in a multi-biobank, meta-analytic setting. Here, we present the GBMI TWAS pipeline, outlining practical considerations for ancestry and tissue specificity, meta-analytic strategies, and open challenges at every step of the framework. We advise conducting ancestry-stratified TWASs using ancestry-specific expression models and meta-analyzing results using inverse-variance weighting, showing the least test statistic inflation. Our work provides a foundation for adding transcriptomic context to biobank-linked GWASs, allowing for ancestry-aware discovery to accelerate genomic medicine.

INTRODUCTION

Population-based or clinical case-based biobanks are key to precision medicine efforts and provide opportunities for genomic research,[1] offering context to deploy genome-wide association studies (GWASs) at scale. Multi-biobank collaborations, like the Global Biobank Meta-analysis Initiative (GBMI), facilitate well-powered, multi-ancestry genetic research and accelerate the understanding of biological mechanisms underlying diseases by *in silico* longitudinal genetic studies and examination of pleiotropy.[2,3]

A key challenge in GWASs is interpreting trait-associated loci with a biological mechanism,[4,5] using methods like colocalization,[6–9] Mendelian randomization (MR),[10–12] and transcriptome-wide association studies (TWASs). TWASs integrate GWASs with expression quantitative trait loci (eQTL) to prioritize gene-trait associations (GTAs) using mediation analysis[13,14] or MR.[15] TWASs involve three steps. First, genetic predictive models of gene expression are trained in the eQTL dataset. Then, genetically regulated expression (GReX) is imputed in the GWAS cohort with individual-level genotypes. Lastly, statistical associations between GReX and trait are estimated.[13–16] TWASs are

This is an open access article under the CC BY license (http://creativecommons.org/licenses/by/4.0/).

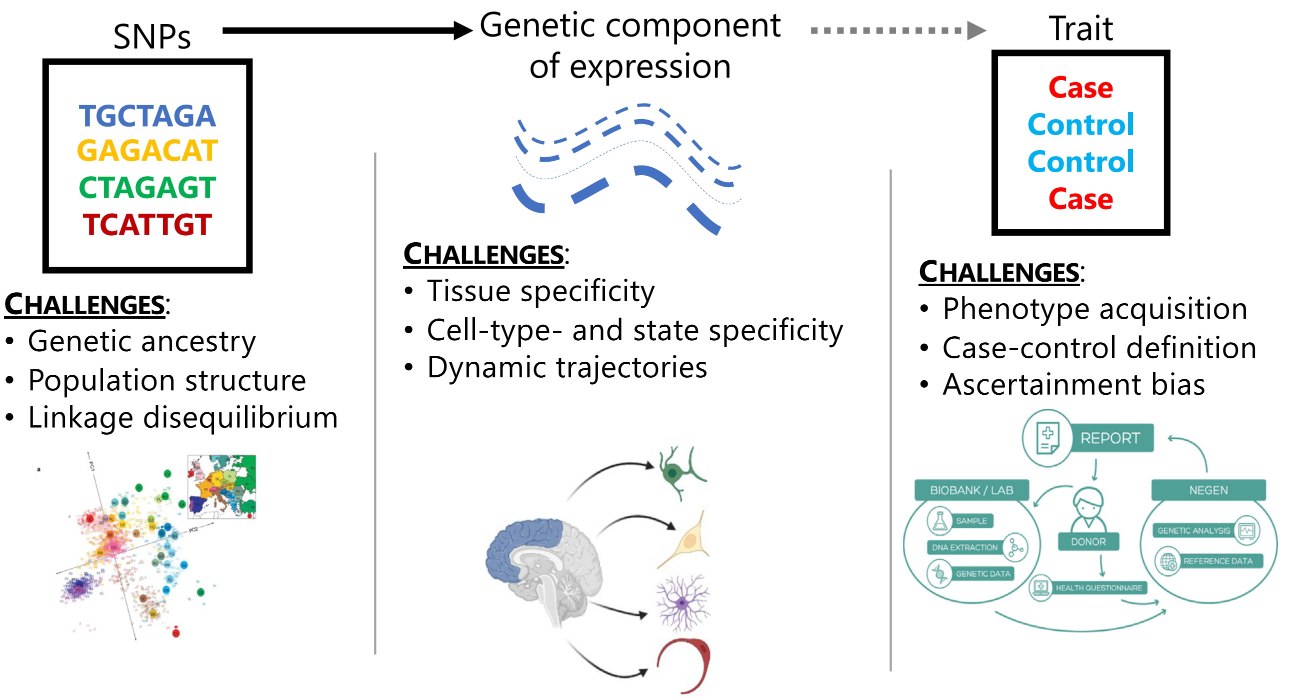

Figure 1. Challenges in multi-ancestry, meta-analytic TWASs

Each level of data in a TWAS introduces a set of challenges: (1) genetics data include confounding from genetic ancestry, population structure and relatedness, and complex linkage disequilibrium patterns, (2) gene expression data introduces context-specific factors, such as tissue-, cell-type-, or cell-state-specific expression, and (3) phenotypic data involve challenges in acquiring and aggregating phenotypes, properly defining controls for phenotypes, and ascertainment and selection bias from non-random sampling.

viable with GWAS summary statistics by estimating the test statistic of the TWAS association using a proper linkage disequilibrium (LD) reference panel.[14] Traditional TWAS methods predict expression using SNPs within 1 Mb of the gene body.[13–16] Recently, methods including strong distal-eQTL signals have shown improved prediction and power to detect GTAs.[17,18] Nonetheless, practical and statistical considerations to accurately prioritize GTAs through TWASs still require methodological improvement.

Along with GWASs, TWASs introduce new challenges by incorporating gene expression[19] (Figure 1). On the genetic level, as in GWASs, disentangling signals from complex LD structure, relatedness, and ancestry requires careful modeling considerations.[20,21] Selection of LD references is specifically important in multi-ancestry settings, like GBMI, as LD structure across ancestry groups differs greatly.[22] Mismatched LD may lead to gene expression models with reduced predictive power, reduced power to detect GTAs, and increased false positives.[23–25] In addition, phenotype acquisition and aggregation are challenging across multiple biobanks with different healthcare, electronic health record, and case-control definitions. However, a challenge specific to TWASs is the integration of gene expression with GWAS signal. Not only is it unclear how to choose an optimal set of genes and tissues best explaining the SNP-trait association, the role of context-specific expression is still being evaluated. For example, dynamic differences in bulk tissue expression due to varied cell types and cell states can provide granularity to GTAs. The impact of these challenges in a meta-analytic framework has not been explored.

Here, we outline a TWAS framework for analyzing multi-ancestry, meta-analytic GWASs across multiple biobanks. We explore practical considerations for all three steps of the TWAS framework (Figures 1 and S1): ancestry specificity of expression models and LD reference panels, meta-analytic techniques for GTA detection, and follow-up analyses for biological context. Given current TWAS frameworks and for analyses using GWAS summary statistics, we recommend training ancestry-specific genetic predictive models of gene expression and conducting TWAS disease mapping by meta-analyzing effect sizes using inverse-variance weighted meta-analysis. Our framework can be applied broadly to study population-wide and ancestry-specific genetic associations with underlying transcriptomic mechanisms.

RESULTS

The GBMI ancestry-wide, meta-analytic TWAS pipeline

We outline the TWAS framework employed in analyses using GWAS summary statistics from the GBMI (Figure S1; details in STAR Methods).[3] First, using joint-tissue imputation (JTI)[26] and multi-omic strategies for TWASs (MOSTWAS),[17] we train ancestry-specific predictive models of gene expression. Next, we identify GTAs using these models and GWAS summary statistics by either multiple-instrumental-variable causal inference or the weighted burden test with an ancestry-matched LD

reference panel for JTI and MOSTWAS models, respectively. This association testing is done for each ancestry group and each biobank. Finally, we perform meta-analysis on the effect sizes across each biobank and each ancestry group using inverse-variance weighting. Lastly, we contextualize GTAs with multiple follow-up analyses.

Expression models are not portable across ancestry groups

GBMI's diversity enables uniquely well-powered studies to detect trait associations in non-European populations. However, optimal TWASs require well-powered training datasets of genetic and tissue-specific gene expression data, which are still lacking for non-European populations. Power to detect GTAs in TWASs is dependent on expression heritability and the predictive power of the expression model.[27] Hence, accurate expression prediction across diverse populations is necessary to ensure that TWAS associations are not restricted to European populations. For the first GBMI TWAS, we restrict analysis to European ancestry populations because of small numbers of non-European ancestry individuals in eQTL reference panels.[28] As sample sizes for eQTL datasets in non-European populations increase, the TWAS pipeline will include expression models for these populations (STAR Methods). We illustrate some challenges in building these expression models across ancestry groups.

In five tissues in GTEx with >70 samples from both European (EUR) and African (AFR) ancestry, we trained EUR- and AFR-specific models using elastic net regularized regression and imputed expression into the aligned (i.e., training and imputation samples have similar ancestries) and misaligned (i.e., training and imputation samples have different ancestries) groups.[29,30] For context, we also built ancestry-unaware models, where EUR and AFR samples were pooled together. Predictive performance was calculated with adjusted 5-fold cross-validation R^2 to account for sample size (STAR Methods). For this analysis, we do not use the JTI or MOSTWAS models, as they either require multi-tissue samples or larger sample sizes than what is available for AFR ancestry individuals, respectively. Both methods borrow from elastic net regression; results from this analysis are applicable to both methods.

Across these tissues, models trained in EUR samples performed, on average, 3–4 times worse (0.02–0.04 difference in median R^2) in AFR samples compared with models trained in AFR samples (Figure 2A; Tables S1–S3). More than 80% of gene models have stronger performance if trained in AFR samples. Distributions of ancestry-aligned and -misaligned adjusted R^2 and percent differences (Figure S2) emphasize that a 0.02–0.04 increase in prediction R^2 is stark. Similar trends hold for ancestry-specific models imputed into down-sampled EUR imputation samples (Figures S3 and S4; Tables S1–S3), consistent with previous simulation and real-world studies[23,25]; here, we considered a randomly selected EUR imputation sample with equal sample size to that of the AFR sample. In fact, we observed that ancestry-specific models imputed into a sample with aligned ancestry showed larger predictive R^2 than ancestry-unaware (individuals of EUR and AFR ancestry in the training sample) models imputed into the same sample (Figure 2B; Table S4), despite increased sample sizes. This observation also holds if we further increase the training sample size by including individuals of other ancestries (Asian, American Indian, and so-called Unknown ancestries) (Figure S5). Not only is this observation in line with recent results from Patel et al. that show differences in causal effect sizes for gene expression across ancestry groups,[31] it also emphasizes the need for ancestry matching in expression prediction and greater recruitment of non-European ancestry patients in eQTL studies.

One reason ancestry-unaware models may perform poorly in AFR samples is because of differences in minor allele frequency (MAF) of predictive SNPs between EUR and AFR ancestry populations. Importantly, this discrepancy is not generally specific to any one ancestry. Rather, ancestry imbalance in training or reference datasets may lead to poor portability of genetic models because of MAF differences. To incorporate common SNPs in both AFR and EUR ancestry populations, we trained ancestry-unaware and ancestry-specific models using SNPs with MAF exceeding various thresholds in both AFR and EUR samples. Excluding SNPs with MAF <0.01 improved predictive performance of ancestry-unaware models across all tissues (Figure S6; Table S5). However, the gap in predictive performance between ancestry-specific and ancestry-unaware models did not decrease when the MAF cutoff was increased (Figure 2B; Table S4). This observation may reflect that dropping ancestry-specific rarer SNPs ignores variants with large ancestry-specific effects on gene expression. Additionally, excluding rare ancestry-specific SNPs does not address the differences in LD across the EUR and AFR samples that lead to different regularization paths and, hence, SNP-gene weights. Addressing the portability of expression models remains an open study direction; methodology that borrows information from functional annotations or across different cell-type- or cell-state-specific contexts may bridge this gap in predictive performance, similar to recent developments in polygenic scores (PGSs)[32] or polygenic transcriptomic risk scores, an analog to PGSs that is constructed using multi-SNP predictors of gene expression.[33,34]

Meta-analytic strategies must be ancestry-aware

Another critical consideration for GBMI involves meta-analysis with GWAS summary statistics. TWASs estimate a GTA by weighting the standardized SNP-trait effect sizes from GWAS summary statistics by SNP-gene weights from the expression models. To account for the correlation between SNPs, an external LD reference panel, like the 1000Genomes Project,[35] is used to estimate the standard error of the TWAS association. Thus, the differences in the reference and in-sample LD in the GWAS cohorts influence the differences in the summary statistics-based TWAS association and TWAS association from direct imputation into individual-level genotypes. Ideally, in-sample LD will give the best estimate of the TWAS standard error, but several biobanks cannot provide this information under specific genetic data sharing and privacy policies. Even departures in LD across subgroups of EUR ancestry populations may influence standard error estimates. In addition, as the estimates of SNP-gene weights are influenced by the LD in the eQTL panel, differences in LD between the eQTL and GWAS panel will also affect the TWAS effect size estimation.

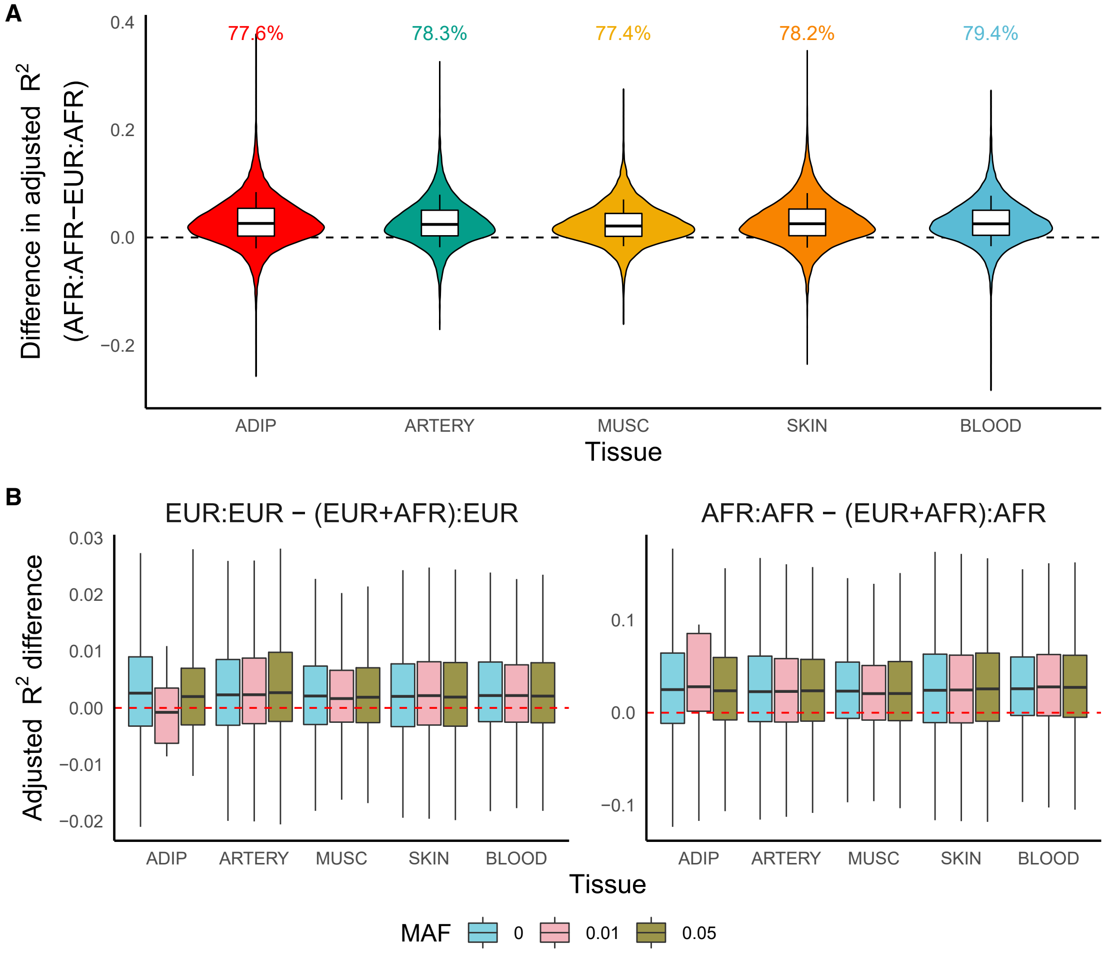

Figure 2. Comparison of predictive performance of expression prediction models across ancestry

(A) Adjusted R^2 difference (y axis) when predicting expression in the AFR imputation sample between models trained in EUR and AFR training samples across tissue (x axis). Proportion of models with improved R^2 using ancestry-aligned models versus ancestry-mismatched models is labeled.

(B) Adjusted R^2 difference between ancestry-specific and ancestry-unaware models imputing into EUR (left) and AFR (right) samples.

As LD patterns differ across ancestry groups,[22] pooling ancestry groups in TWASs may lead to reduced power. We conducted TWASs for asthma risk using ancestry-unaware and EUR- and AFR-specific models of whole blood expression (4,782 genes; see STAR Methods). Ancestry-specific TWAS *Z* scores across EUR and AFR ancestry groups were not strongly correlated ($r = 0.11$), potentially because of differences in sample size and eQTL and GWAS architecture (Figures 3A, S7, and S8).[36,37] For genes with $p < 2.5 \times 10^{-6}$ in either EUR or AFR ancestry groups, both SNP-gene effects and corresponding standardized effect sizes for these SNPs from ancestry-specific meta-analyzed GWASs show very low correlation (Figure S9). These results reinforce the lessons from the low cross-ancestry group correlations of TWAS *Z* scores and suggest that model training and association testing should be conducted within ancestry groups.

In fact, we detected only two genes that had a significant association with asthma in both EUR and AFR ancestry groups with $p < 2.5 \times 10^{-6}$. One of these genes, *DFFA*, has been implicated with asthma risk through GWASs and colocalization in EURs.[38] However, the TWAS associations across EUR and AFR ancestry groups went in opposite directions using blood tissue. In the other four tissues explored, *DFFA* TWAS associations did not reach transcriptome-wide significance, but effect directions were generally concordant (Figure S10). In blood, lead local-eQTLs (within 1 Mb) of *DFFA* are in opposite directions, though only nominally significant at $p < 0.05$ (Figure S11). Although they are within 60 kb, the lead eQTLs for DFFA across AFR (rs263526) and EUR (rs903916) ancestry groups are not in LD ($R^2 = 3 \times 10^{-4}$ in AFR, 0.0072 in EUR). The GWAS effect sizes of SNPs local to *DFFA* do not show large deviations in effect direction and are only nominally significant as well (Figure S11). These differences in TWAS associations across ancestry motivate careful consideration of meta-analytic strategy to avoid biasing cross-ancestry associations toward cohorts with larger sample sizes, which still tend to be predominantly of EUR ancestry.

We investigated five meta-analytic strategies: meta-analyzing across ancestry-specific, per-biobank GWAS summary statistics

CellPress
OPEN ACCESS

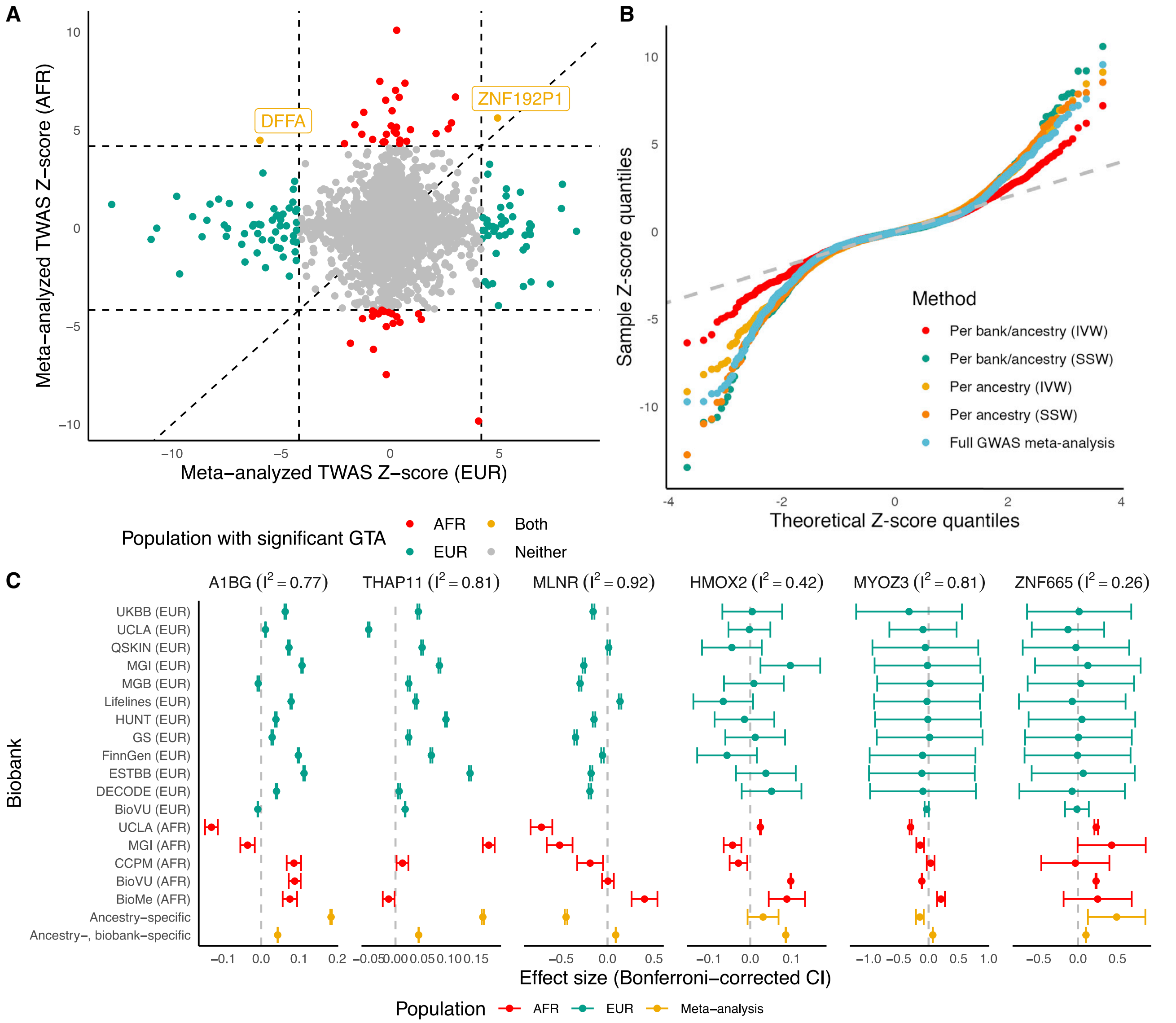

Figure 3. Comparison of meta-analytic strategies for multi-biobank, multi-ancestry TWASs

(A) Per-ancestry meta-analyzed TWAS scores across EUR (x axis) versus AFR ancestry (y axis). The dotted lines indicate $p < 2.5\times10^{-6}$ with a 45-degree line for reference. Points are colored by which ancestry population the TWAS association meets $p < 2.5\times10^{-6}$.

(B) QQ-plot of TWAS Z scores , colored by meta-analytic strategies. Per ancestry refers to TWAS meta-analysis across meta-analyzed ancestry-specific GWAS summary statistics. Per bank/per ancestry refers to TWAS meta-analysis using all biobank- and ancestry-specific GWAS summary statistics.

(C) Effect sizes and Bonferroni-corrected confidence intervals (CIs) for TWAS associations across 17 individual biobanks (EUR in teal, AFR in red) and two IVW meta-analysis strategies (in yellow) for five representative genes. The Higgins-Thompson I^2 statistic is provided.

using (1) inverse-variance weighting (IVW) and (2) effective sample size weighting (SSW), meta-analyzing across ancestry-specific meta-analyzed GWAS summary statistics using (3) IVW and (4) SSW, and (5) performing a TWAS using ancestry-unaware models and meta-analyzed GWAS summary statistics across EUR and AFR ancestry groups (STAR Methods). QQ-plots in Figure 3B show earlier departure of Z scores from the QQ-line for SSW meta-analyzed Z scores and the ancestry-unaware strategy, suggesting type I error inflation. This observation is supported with estimates of test statistic bias and inflation, with the largest estimated bias and inflation for these SSW and ancestry-unaware methods.[39] IVW strategies show similar levels of inflation, with IVW meta-analysis across ancestry-specific meta-analyzed GWAS summary statistics showing minimal bias (Figure S12). More naive SSW meta-analysis and ancestry-unaware methods bias toward the larger EUR cohorts, whereas Z scores from the IVW methods showed positive correlations with Z scores from AFR cohorts (Figure S8).

However, it is unclear whether ancestry-specific IVW meta-analysis to the per-biobank level is necessary. As shown in

Figure S13, Z scores from these two IVW methods are moderately positively correlated ($r = 0.51$ across 4,152 tests), with this correlation increasing when we consider genes with nominally significant Z scores for both strategies ($r = 0.70$, 564 tests). We observed that top associations across these IVW meta-analyses often had high degrees of heterogeneity in effect size across biobanks, measured by the Higgins-Thompson I^2 statistic[40] (Figures 3C and S14). When using the weighted burden test, the standard error and confidence interval widths are functions of only SNP-gene weights and the reference LD matrix; GWAS sample size does not have an effect on the standard error. One gene, *A1BG*, that showed directionally concordant associations across both IVW strategies had large heterogeneity across the participating cohorts ($I^2 = 0.77$). In fact, the cross-biobank heterogeneity is often larger than the cross-ancestry heterogeneity for TWAS associations of *A1BG*. *ZNF665*, another gene with concordant associations across IVW strategies, showed a low heterogeneity in the per-biobank effect sizes ($I^2 = 0.26$). However, genes with discordant associations across IVW strategies showed varied patterns. Two illustrative examples are *MLNR* and *MYOZ3*, both with large degrees of test statistic heterogeneity ($I^2 = 0.91$ and 0.82, respectively). Across the two IVW strategies, effect sizes are in opposite directions, possibly due to large standard error differences across the ancestry-specific per-biobank associations. A thorough investigation of the power and false discovery rates of these meta-analysis strategies through simulations is necessary. Methods that incorporate the per-biobank uncertainty must be explored to increase power and properly leverage the large sample sizes of GBMI.[41–43] A recent method called METRO is a promising route for multi-ancestry TWASs because it combines multiple ancestry-specific expression models in a joint likelihood-based inference framework and accounts for uncertainty in the prediction models across the various ancestry groups.[44]

In addition to multi-ancestry populations, analyzing genetic data from individuals of admixed ancestry is also an open area of study. In this analysis, we have used the 1000Genomes AFR LD reference panel as an estimate of the LD for AFR ancestry samples from each biobank. However, most of GBMI's AFR ancestry populations are of admixed ancestry (e.g., African Americans or African British). A single LD reference panel of AFR ancestry may not reflect the genetic diversity in these admixed populations of AFR and EUR ancestries.[45] In admixed populations, using local ancestry estimates aids in better characterization of heritability of complex traits and more accurate mapping of genetic associations, especially eQTLs.[46] Incorporating ancestry-specific allelic effects on gene expression, estimated from phased genotypes and local ancestry inference, into TWASs may lead to increased power and should be explored. However, errors in ancestry inference and heterogeneity in ancestry-specific effect may pose challenges for these methodological extensions.

Follow-up tests provide biological and clinical context to TWAS GTAs

TWAS GTAs identified using GWAS summary statistics are subject to several factors that may lead to false positives. We implement several follow-up tests to provide context to TWAS-identified GTAs. First, a TWAS GTA could attain significance due only to strong SNP-trait associations from the underlying GWAS. To quantify the significance of the GTA conditional on the SNP-trait effects at the locus, we perform a permutation test by permuting the SNP-gene weights from the expression model to generate a null distribution (STAR Methods). Comparing the original TWAS Z score to this null distribution assesses how much signal is added by the expression given the specific GWAS architecture of the locus. This permutation test is highly conservative and prioritizes only associations already significant in the standard TWAS GTA detection.[14]

Next, gene expression models for genes in adjacent genomic windows may be built from overlapping SNPs or SNPs in strong LD. When TWASs detect GTAs in overlapping genomic regions, we apply probabilistic fine-mapping using FOCUS[47] to estimate a 90% credible set of genes to explain the observed association signal in a given tissue (STAR Methods). However, the current iteration of FOCUS has limitations. Priors for the correlation matrix between GReX of overlapping genes are dependent on SNP LD reference panels. Thus, fine-mapping in trans-ancestry settings is difficult, though recent additions to the FOCUS framework, called multi-ancestry FOCUS (MA-FOCUS), account for differences in genetic architecture across the study sample.[48] Another challenge for gene-level fine-mapping in multi-tissue TWASs is distinguishing between overlapping signals across tissues. Primarily because of cross-cell-type variation in expression levels and eQTL architecture, TWASs may prioritize genes in multiple tissues that are overrepresented by the same underlying causal cell types.[19] This multi-tissue gene prioritization extends to fine-mapping overlapping TWAS signals across tissue, as priors for FOCUS are not tissue dependent; extracting posterior signal that is biologically consistent and meaningful without allowing the prior to dominate is challenging.

The GBMI TWAS pipeline incorporates gene expression models using MOSTWAS, which prioritizes distal-eQTLs by testing their mediation effect through local molecular features (STAR Methods). For genes with models trained with MOSTWAS and associated with the trait at transcriptome-wide significance, we test the additional association signal from the distal-SNPs using an added-last test, analogous to a group-added-last test in linear regression.[17] This test prioritizes sets of genomic or epigenomic features that mediate the predicted distal-eQTLs for subsequent study of upstream, tissue-specific regulation of GTAs. In one application of MOSTWAS, one prioritized functional hypothesis was experimentally validated *in vitro*.[49] As distal-eQTLs are more likely to be tissue or cell type specific,[50] the association signal from these distal-eQTLs could also be leveraged in cross-tissue fine-mapping strategies.

Lastly, TWASs suffer from severely reduced power and inflated false positives in the presence of SNP horizontal pleiotropy.[51] We encourage estimating the degree of and accounting for SNP pleiotropy using LDA-MR-Egger[52] or PMR-Egger,[53] especially in settings with individual-level GWAS genotypes. Applications for these methods using GWAS summary statistics reveal some inflation of standard errors,[54] suggesting the need for further evaluation and development of summary statistics-based methods.

CellPress
OPEN ACCESS

Biobanks enable GReX-PheWAS for biological context

Biobanks aggregated in GBMI provide a rich catalog of phenotypes for analysis, with phenotype codes (phecodes) aggregated from ICD codes classified into clinically relevant categories.[55] This phenotype catalog enables phenome-wide association studies (PheWASs) as a complement to GWASs by both replicating GWAS associations and providing a larger set of trait associations with GWAS variants. To follow up on novel TWAS-prioritized genes, the PheWAS framework can be expanded to the GReX level in a similar analysis: GReX-level phenome-wide association study (GReX-PheWAS), similar to the PredixVU database.[56–59] Not only do these analyses replicate and detect new TWAS associations, they can also point to groups of phenotypes that show enrichments for trait associations for the gene of interest.

We briefly illustrate an example of a GReX-PheWAS using three genes (Figures 4, S15, and S16; Table S6): *TAF7*, a novel gene in our TWAS, and *ILRAP18* and *TMEM258*, two genes previously implicated through GWASs.[60–64] These genes were prioritized from European-specific TWASs for asthma risk from the flagship GBMI project using lung tissue expression (101,311 cases and 1,118,682 controls): *TAF7* (MOSTWAS model), *IL18RAP* (JTI model), and *TMEM258* (JTI model). European-specific TWAS meta-analysis for asthma detected a negative association with *TAF7* cis-GReX, a gene that did not intersect a GWAS-significant locus. In TWAS follow-up tests, *TAF7* passed permutation testing and was estimated in the 90% credible set at the genomic locus via FOCUS with posterior inclusion probability 1. As the clinically relevant associations for *TAF7* lung GReX are not characterized, we employed a GReX-PheWAS in UKBB European ancestry GWAS summary statistics across 731 traits and diseases with sample sizes greater than 100,000, grouped into nine categories (Figure 4 and STAR Methods). We see enrichments for phenotypes of the hematopoietic and musculoskeletal groups (Figure 4A) with hypothyroidism and chronic laryngitis as the top phenotype associations (Figure 4B; Table S6). The curved nature of the Miami plots in Figures 4 and S14 is only because we plot *Z* scores in decreasing absolute value within phecode groups. These phenotypes include multiple inflammations of organs (e.g., laryngitis, osteitis, meningitis, etc.). We also detected several associations with related respiratory diseases and traits. Similarly, for the two previously implicated genes, we find enrichments for respiratory and hematopoietic GTAs for *IL-RAP18* and across multiple categories for *TMEM258*, consistent with the categorized functions and associations of these genes (Figures S15 and S16; Table S6). GBMI's wide roster of phenotypes enables the GReX-PheWAS to add biological and clinical context to novel TWAS associations.

GReX-PheWAS, despite its utility, shares the challenges of the PheWAS. Phenotypes within and across groups may be correlated, leading to a series of dependent tests. Thus, simple adjustments of multiple testing burden may not be appropriate, and methods that account for correlation between phenotypes, like permutation tests, may be more applicable.[55,65–67] In addition, covariate adjustments in expression models built for disease-specific analyses may not be generalizable for multiple phenotypes. Most population-based clinical biobanks lack comprehensive clinical and lifestyle information of the individuals because of gaps in electronic health records. Phenotype groupings may also be deceptive: as most biobanks follow ICD coding that groups traits and diseases by body systems, GReX-PheWAS enrichments for a given group may not reflect shared genetic pathways across body systems. In addition, case-control selection may not be optimal because of differences in exclusion criteria.[68] Despite these limitations in phenotype acquisition, recent methods focusing on identifying shared genetic architecture among multiple phenotypes in a phenome-wide approach highlight the advantages of GReX-PheWASs.[68]

DISCUSSION

Here, we provide a framework for TWASs in a multi-biobank setting across many ancestry groups. In general, for TWASs using GWAS summary statistics, we recommend a meta-analytic approach across both ancestry groups and individual cohorts. As multi-tissue transcriptomic reference panels and eQTL resources increase in sample size for populations not of European ancestry (e.g., African, Hispanic/Latin American, and East and South Asian ancestries that are represented broadly by GBMI), we will extend our TWAS analyses to interrogate underlying transcriptomic mechanisms underlying the complex traits studied with GBMI. We outline several methodological gaps to be addressed in the future: (1) training expression models that are portable across ancestry groups, (2) limiting false discovery in TWASs by properly modeling differences in LD across ancestry groups, (3) incorporating uncertainty within and heterogeneity across biobanks to boost TWAS meta-analytic power, and (4) contextualizing TWAS GTAs through follow-up testing, probabilistic fine-mapping across ancestry groups and expression contexts, and GReX-PheWASs.

Along with the discussed issues with current TWAS methodology, tissue-specific expression may not provide sufficient granularity needed to discover trait-relevant biological mechanisms. Recent methods that study the mediation of the SNP-trait relationship by cell-type heterogeneity show that cell types and cell states are influenced by genetics and predict complex traits, and modeling these directly may lead to improved power in detecting trait associations.[26,69–72] Single-cell eQTL datasets can be integrated with GWASs to identify context-specific expression pathways that are disease related. Incorporating single-cell expression data into a predictive model will require improved methodology that models cell identity as a spectrum.[73,74] Multiomic approaches incorporating functional data with TWASs may better model the flow of biological information in a biologically interpretable fashion.[17,75,76]

Despite the limitations of this suite of methods, TWASs continue to be a useful tool for interpreting GWAS associations and independently discovering genetic associations mediated by gene expression. More sophisticated integrative computational and experimental tools to complement improved TWASs and GWASs to understand the biology underlying health and disease need to be developed. Most importantly, reference eQTL data from individuals of non-European ancestry needs to be collected at parity with those of European ancestry individuals.

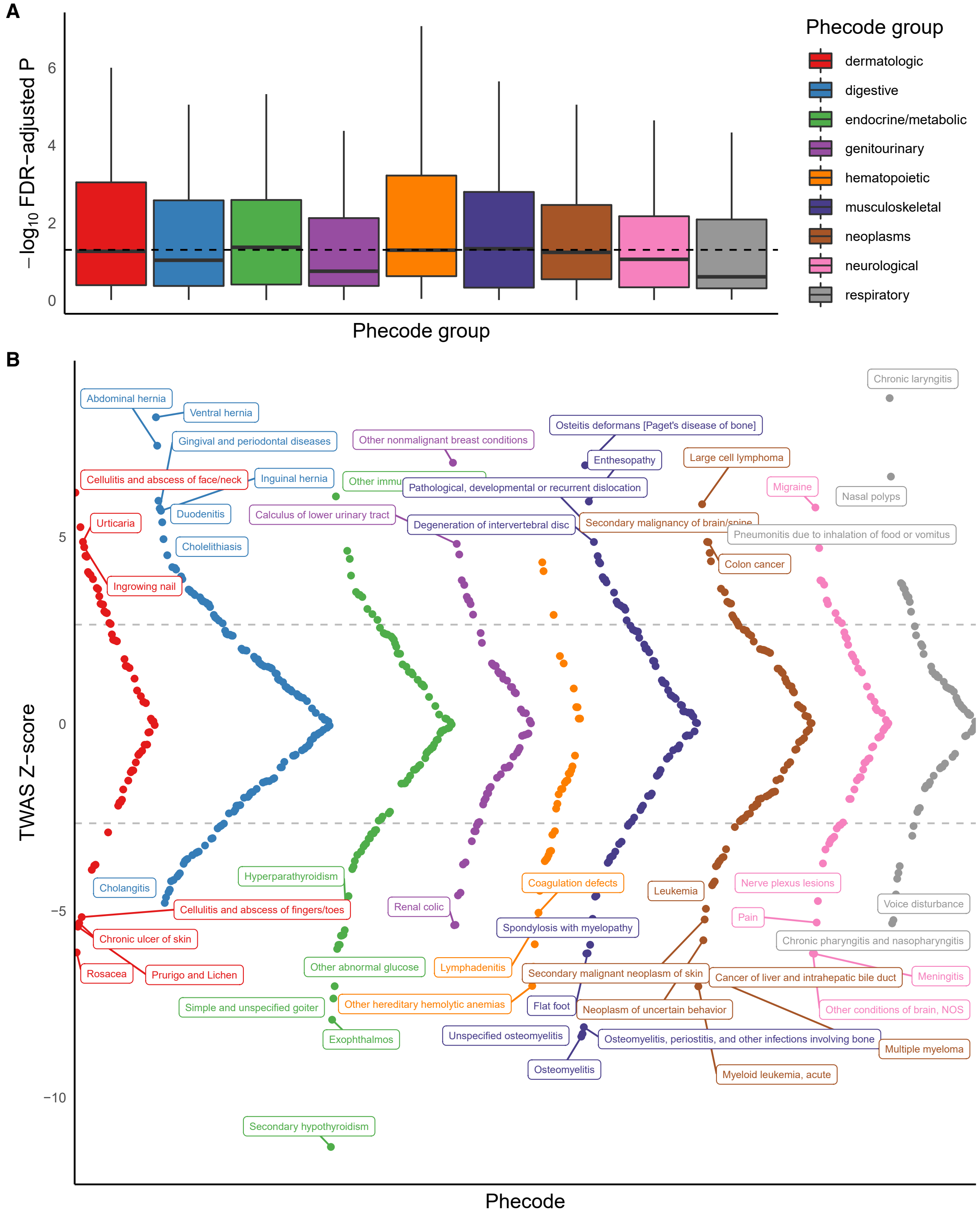

Figure 4. GReX-PheWAS for categorizing phenome-wide associations for TAF7 genetically regulated expression in UKBB

(A) $-\log_{10}$ Benjamini-Hochberg FDR-adjusted p values of GTAs (y axis) across nine phenotype groups (x axis). Dotted line shows FDR-adjusted p = 0.05.

(B) Miami plot of TWAS *Z* scores (y axis) across phenotypes (x axis), colored by phecode group. Dotted line shows Benjamini-Hochberg FDR-corrected significance, and phenotypes are labeled if the association passes Bonferroni correction.

CellPress
OPEN ACCESS

Limitations of the study

We conclude with a few limitations of our evaluations of TWASs. We use eQTL data from GTEx to assess portability of expression prediction models, which have limited AFR ancestry sample sizes. These analyses should also be performed in larger cohorts of non-European populations, even though our results reflect previous evaluations. In addition, we evaluate only traditional TWAS methods. Though other methods are similar to these first TWAS methods, other frameworks may be better equipped to harmonize expression prediction and GTA mapping across ancestry groups. Lastly, we present only fixed effect meta-analyses. Meta-analysis with random effects models may prove to be more powerful.

CONSORTIA

Kuan-Han H. Wu, Humaira Rasheed, Kristin Tsuo, Ying Wang, Huiling Zhao, Shinichi Namba, Ida Surakka, Brooke N. Wolford, Valeria Lo Faro, Esteban A. Lopera-Maya, Kristi Läll, Marie-Julie Favé, Sinéad B. Chapman, Juha Karjalainen, Mitja Kurki, Maasha Mutaamba, Juulia Partanen, Ben M. Brumpton, Sameer Chavan, Tzu-Ting Chen, Michelle Daya, Yi Ding, Yen-Chen A. Feng, Christopher R. Gignoux, Sarah E. Graham, Whitney E. Hornsby, Nathan Ingold, Ruth Johnson, Triin Laisk, Kuang Lin, Jun Lv, Iona Y. Millwood, Priit Palta, Anita Pandit, Michael H. Preuss, Unnur Thorsteinsdottir, Jasmina Uzunovic, Matthew Zawistowski, Xue Zhong, Archie Campbell, Kristy Crooks, Geertruida H. de Bock, Nicholas J. Douville, Sarah Finer, Lars G. Fritsche, Christopher J. Griffiths, Yu Guo, Karen A. Hunt, Takahiro Konuma, Riccardo E. Marioni, Jansonius Nomdo, Snehal Patil, Nicholas Rafaels, Anne Richmond, Jonathan A. Shortt, Peter Straub, Ran Tao, Brett Vanderwerff, Kathleen C. Barnes, Marike Boezen, Zhengming Chen, Chia-Yen Chen, Judy Cho, George Davey Smith, Hilary K. Finucane, Lude Franke, Andrea Ganna, Tom R. Gaunt, Tian Ge, Hailiang Huang, Jennifer Huffman, Jukka T. Koskela, Clara Lajonchere, Matthew H. Law, Liming Li, Cecilia M. Lindgren, Ruth J.F. Loos, Stuart MacGregor, Koichi Matsuda, Catherine M. Olsen, David J. Porteous, Jordan A. Shavit, Harold Snieder, Richard C. Trembath, Judith M. Vonk, David Whiteman, Stephen J. Wicks, Cisca Wijmenga, John Wright, Xiang Zhou, Philip Awadalla, Michael Boehnke, Daniel H. Geschwind, Caroline Hayward, Kristian Hveem, Eimear E. Kenny, Yen-Feng Lin, Reedik Mägi, Hilary C. Martin, Sarah E. Medland, Yukinori Okada, Aarno V. Palotie, Serena Sanna, Jordan W. Smoller, Kari Stefansson, David A. van Heel, Robin G. Walters, Sebastian Zöllner, Biobank Japan, BioMe, BioVU, Canadian Partnership for Tomorrow's Health/Ontario Health Study, China Kadoorie Biobank Collaborative Group, Colorado Center for Personalized Medicine, deCODE Genetics, Estonian Biobank, FinnGen, Generation Scotland, Genes & Health, LifeLines, Mass General Brigham Biobank, Michigan Genomics Initiative, QIMR Berghofer Biobank, Taiwan Biobank, The HUNT Study, UCLA ATLAS Community Health Initiative, UK Biobank, Alicia R. Martin, Cristen J. Willer, Mark J. Daly, and Benjamin M. Neale.

STAR★METHODS

Detailed methods are provided in the online version of this paper and include the following:

- KEY RESOURCES TABLE
- RESOURCE AVAILABILITY
  - Lead contact
  - Materials availability
  - Data and code availability
- EXPERIMENTAL MODEL AND SUBJECT DETAILS
- METHOD DETAILS
  - The GBMI TWAS pipeline
  - Analysis of ancestry-specific and -unaware models
- QUANTIFICATION AND STATISTICAL ANALYSIS
  - Strategies for meta-analysis
  - GReX-level phenome-wide association studies (GReX-PheWAS)

SUPPLEMENTAL INFORMATION

Supplemental information can be found online at https://doi.org/10.1016/j.xgen.2022.100180.

ACKNOWLEDGMENTS

We thank Mark Daly for helpful comments and advice during the internal revision process and Nicholas Mancuso, Michael Love, Yun Li, and Achal Patel for their thoughtful discussion during the research process. We would like to thank the organizing committee of the International Common Disease Alliance for intellectual contributions on the setup of the GBMI as a nascent activity to the larger effort. We would like to thank Daniel King from the Hail team and Sam Bryant from the Stanley Center Data Management team at the Broad Institute for helping with the Google bucket setup and data sharing and Bethany Klunder from the University of Michigan Medical school for helping with the paper submission. B.P. was partially supported by NIH awards R01 HG009120, R01 MH115676, R01 CA251555, R01 AI153827, R01 HG006399, R01 CA244670, and U01 HG011715. E.R.G. is supported by the National Institutes of Health (NIH) grants NHGRI R35HG010718, NHGRI R01HG011138, NIA AG068026, and NIGMS R01GM140287. N.J.C. is supported by U01HG009086. W.Z. was supported by the National Human Genome Research Institute of the National Institutes of Health under award numbers T32HG010464 and K99HG012222-01.

AUTHOR CONTRIBUTIONS

Conceptualization, A.B. and J.H.; methodology, A.B., J.H., D.Z., E.R.G., B.P., and N.J.C.; software, A.B., J.H., D.Z., E.R.G., B.P., and N.J.C.; validation, A.B., J.H., and D.Z.; formal analysis, A.B. and J.H.; investigation, all authors; resources, all authors; data curation, W.Z. and M.K.; writing – original draft, A.B. and J.H.; writing – review & editing, all authors; visualization, A.B. and J.H.; supervision, E.G., B.P., and N.J.C.; project administration, A.B., J.H., E.R.G., B.P., and N.J.C.; funding acquisition, all authors.

DECLARATION OF INTERESTS

The authors declare no competing interests.

INCLUSION AND DIVERSITY

We worked to ensure gender balance in the recruitment of human subjects. We worked to ensure ethnic or other types of diversity in the recruitment of human subjects. One or more of the authors of this paper self-identifies as an underrepresented ethnic minority in science. One or more of the authors of this paper self-identifies as a member of the LGBTQ+ community. One or more of the authors of this paper received support from a program designed to increase minority representation in science. While citing references scientifically relevant for this work, we also actively worked to promote gender balance in our reference list. The author list of this paper includes contributors from the

location where the research was conducted who participated in the data collection, design, analysis, and/or interpretation of the work.

Received: November 28, 2021
Revised: August 9, 2022
Accepted: September 1, 2022
Published: October 12, 2022

REFERENCES

1. Abul-Husn, N.S., and Kenny, E.E. (2019). Personalized medicine and the power of electronic health records. Cell *177*, 58–69.
2. Swede, H., Stone, C.L., and Norwood, A.R. (2007). National population-based biobanks for genetic research. Genet. Med. *9*, 141–149.
3. Zhou, W., Kanai, M., Wu, K.-H.H., Humaira, R., Tsuo, K., Hirbo, J.B., Wang, Y., Bhattacharya, A., Zhao, H., Namba, S., et al. (2021). Global Biobank Meta-analysis Initiative: powering genetic discovery across human diseases. Preprint at medRxiv *27*. https://doi.org/10.1101/2021.11.19.21266436.
4. Gallagher, M.D., and Chen-Plotkin, A.S. (2018). The post-GWAS era: from association to function. Am. J. Hum. Genet. *102*, 717–730.
5. Wijmenga, C., and Zhernakova, A. (2018). The importance of cohort studies in the post-GWAS era. Nat. Genet. *50*, 322–328.
6. Giambartolomei, C., Vukcevic, D., Schadt, E.E., Franke, L., Hingorani, A.D., Wallace, C., and Plagnol, V. (2014). Bayesian test for colocalisation between pairs of genetic association studies using summary statistics. PLoS Genet. *10*, e1004383.
7. Giambartolomei, C., Zhenli Liu, J., Zhang, W., Hauberg, M., Shi, H., Boocock, J., Pickrell, J., Jaffe, A.E., and CommonMind Consortium; and Roussos, P. (2018). A Bayesian framework for multiple trait colocalization from summary association statistics. Bioinformatics *34*, 2538–2545.
8. Gleason, K.J., Yang, F., Pierce, B.L., He, X., and Chen, L.S. (2020). Primo: integration of multiple GWAS and omics QTL summary statistics for elucidation of molecular mechanisms of trait-associated SNPs and detection of pleiotropy in complex traits. Genome Biol. *21*, 236.
9. He, X., Fuller, C.K., Song, Y., Meng, Q., Zhang, B., Yang, X., and Li, H. (2013). Sherlock: detecting gene-disease associations by matching patterns of expression QTL and GWAS. Am. J. Hum. Genet. *92*, 667–680.
10. Hauberg, M.E., Zhang, W., Giambartolomei, C., Franzén, O., Morris, D.L., Vyse, T.J., Ruusalepp, A., CommonMind Consortium; Schadt, E.E., Björkegren, J.L.M., and Roussos, P. (2017). Large-scale identification of common trait and disease variants affecting gene expression. Am. J. Hum. Genet. *101*, 157–894.
11. Pavlides, J.M.W., Zhu, Z., Gratten, J., McRae, A.F., Wray, N.R., and Yang, J. (2016). Predicting gene targets from integrative analyses of summary data from GWAS and eQTL studies for 28 human complex traits. Genome Med. *8*, 84.
12. Smith, G.D., and Ebrahim, S. (2003). 'Mendelian randomization': can genetic epidemiology contribute to understanding environmental determinants of disease? Int. J. Epidemiol. *32*, 1–22.
13. Gamazon, E.R., Wheeler, H.E., Shah, K.P., Mozaffari, S.V., Aquino-Michaels, K., Carroll, R.J., Eyler, A.E., Denny, J.C., GTEx Consortium; Cox, N.J., and Im, H.K. (2015). A gene-based association method for mapping traits using reference transcriptome data. Nat. Genet. *47*, 1091–1098.
14. Gusev, A., Ko, A., Shi, H., Bhatia, G., Chung, W., Penninx, B.W.J.H., Jansen, R., de Geus, E.J.C., Boomsma, D.I., Wright, F.A., et al. (2016). Integrative approaches for large-scale transcriptome-wide association studies. Nat. Genet. *48*, 245–252.
15. Zhang, Y., Quick, C., Yu, K., Barbeira, A., GTEx Consortium; Pique-Regi, R., Kyung Im, H., and Wen, X. (2020). PTWAS: investigating tissue-relevant causal molecular mechanisms of complex traits using probabilistic TWAS analysis. Genome Biol. *21*, 232–326.
16. Barbeira, A.N., Dickinson, S.P., Bonazzola, R., Zheng, J., Wheeler, H.E., Torres, J.M., Torstenson, E.S., Shah, K.P., Garcia, T., Edwards, T.L., et al. (2018). Exploring the phenotypic consequences of tissue specific gene expression variation inferred from GWAS summary statistics. Nat. Commun. *9*, 1825–1920.
17. Bhattacharya, A., Li, Y., and Love, M.I. (2021). MOSTWAS: multi-omic strategies for transcriptome-wide association studies. PLoS Genet. *17*, e1009398.
18. Luningham, J.M., Chen, J., Tang, S., De Jager, P.L., Bennett, D.A., Buchman, A.S., and Yang, J. (2020). Bayesian genome-wide TWAS method to leverage both cis- and trans-eQTL information through summary statistics. Am. J. Hum. Genet. *107*, 714–726.
19. Wainberg, M., Sinnott-Armstrong, N., Mancuso, N., Barbeira, A.N., Knowles, D.A., Golan, D., Ermel, R., Ruusalepp, A., Quertermous, T., Hao, K., et al. (2019). Opportunities and challenges for transcriptome-wide association studies. Nat. Genet. *51*, 592–599.
20. Mbatchou, J., Barnard, L., Backman, J., Marcketta, A., Kosmicki, J.A., Ziyatdinov, A., Benner, C., O'Dushlaine, C., Barber, M., Boutkov, B., et al. (2020). Computationally efficient whole genome regression for quantitative and binary traits. Preprint at bioRxiv. https://doi.org/10.1101/2020.06.19.162354.
21. Zhou, W., Nielsen, J.B., Fritsche, L.G., Dey, R., Gabrielsen, M.E., Wolford, B.N., LeFaive, J., VandeHaar, P., Gagliano, S.A., Gifford, A., et al. (2018). Efficiently controlling for case-control imbalance and sample relatedness in large-scale genetic association studies. Nat. Genet. *50*, 1335–1341.
22. Shifman, S., Kuypers, J., Kokoris, M., Yakir, B., and Darvasi, A. (2003). Linkage disequilibrium patterns of the human genome across populations. Hum. Mol. Genet. *12*, 771–776.
23. Bhattacharya, A., García-Closas, M., Olshan, A.F., Perou, C.M., Troester, M.A., and Love, M.I. (2020). A framework for transcriptome-wide association studies in breast cancer in diverse study populations. Genome Biol. *21*, 42.
24. Geoffroy, E., Gregga, I., and Wheeler, H.E. (2020). Population-matched transcriptome prediction increases TWAS discovery and replication rate. iScience *23*, 101850.
25. Keys, K.L., Mak, A.C.Y., White, M.J., Eckalbar, W.L., Dahl, A.W., Mefford, J., Mikhaylova, A.V., Contreras, M.G., Elhawary, J.R., Eng, C., et al. (2020). On the cross-population generalizability of gene expression prediction models. PLoS Genet. *16*, e1008927.
26. Zhou, D., Jiang, Y., Zhong, X., Cox, N.J., Liu, C., and Gamazon, E.R. (2020). A unified framework for joint-tissue transcriptome-wide association and Mendelian randomization analysis. Nat. Genet. *52*, 1239–1246.
27. Cao, C., Ding, B., Li, Q., Kwok, D., Wu, J., and Long, Q. (2021). Power analysis of transcriptome-wide association study: implications for practical protocol choice. PLoS Genet. *17*, e1009405.
28. The GTEx Consortium; Barbeira, A.N., Bonazzola, R., Brown, A., Castel, S.E., Jo, B., Kasela, S., Kim-Hellmuth, S., Liang, Y., Oliva, M., et al. (2020). The GTEx Consortium atlas of genetic regulatory effects across human tissues. Science *369*, 1318–1330.
29. Friedman, J., Hastie, T., and Tibshirani, R. (2010). Regularization paths for generalized linear models via coordinate descent. J. Stat. Softw. *33*, 1–22.
30. Yang, J., Lee, S.H., Goddard, M.E., and Visscher, P.M. (2011). GCTA: a tool for genome-wide complex trait analysis. Am. J. Hum. Genet. *88*, 76–82.
31. Patel, R.A., Musharoff, S.A., Spence, J.P., Pimentel, H., Tcheandjieu, C., Mostafavi, H., Sinnott-Armstrong, N., Clarke, S.L., Smith, C.J., Program, V.M.V., et al. (2022). Genetic Interactions Drive Heterogeneity in Causal Variant Effect Sizes for Gene Expression and Complex Traits. Preprint at bioRxiv. https://doi.org/10.1101/2021.12.06.471235.
32. Amariuta, T., Ishigaki, K., Sugishita, H., Ohta, T., Koido, M., Dey, K.K., Matsuda, K., Murakami, Y., Price, A.L., Kawakami, E., et al. (2020). Improving the trans-ancestry portability of polygenic risk scores by prioritizing variants in predicted cell-type-specific regulatory elements. Nat. Genet. *52*, 1346–1354.

CellPress
OPEN ACCESS

33. Hu, X., Qiao, D., Kim, W., Moll, M., Balte, P.P., Lange, L.A., Bartz, T.M., Kumar, R., Li, X., Yu, B., et al. (2022). Polygenic transcriptome risk scores for COPD and lung function improve cross-ethnic portability of prediction in the NHLBI TOPMed program. Am. J. Hum. Genet. *109*, 857–870.

34. Liang, Y., Pividori, M., Manichaikul, A., Palmer, A.A., Cox, N.J., Wheeler, H.E., and Im, H.K. (2022). Polygenic transcriptome risk scores (PTRS) can improve portability of polygenic risk scores across ancestries. Genome Biol. *23*, 23.

35. 1000 Genomes Project Consortium; Brooks, L.D., Garrison, E.P., Durbin, R.M., McCarthy, S., Abecasis, G.R., Clark, A.G., Donnelly, P., Eichler, E.E., Flicek, P., et al. (2015). A global reference for human genetic variation. Nature *526*, 68–74.

36. Shang, L., Smith, J.A., Zhao, W., Kho, M., Turner, S.T., Mosley, T.H., Kardia, S.L.R., and Zhou, X. (2020). Genetic architecture of gene expression in European and african Americans: an eQTL mapping study in GENOA. Am. J. Hum. Genet. *106*, 496–512.

37. Wyss, A.B., Sofer, T., Lee, M.K., Terzikhan, N., Nguyen, J.N., Lahousse, L., Latourelle, J.C., Smith, A.V., Bartz, T.M., Feitosa, M.F., et al. (2018). Multiethnic meta-analysis identifies ancestry-specific and cross-ancestry loci for pulmonary function. Nat. Commun. *9*, 2976–3015.

38. Vicente, C.T., Revez, J.A., and Ferreira, M.A.R. (2017). Lessons from ten years of genome-wide association studies of asthma. Clin. Transl. Immunology *6*, e165.

39. van Iterson, M., van Zwet, E.W., and Heijmans, B.T.; BIOS Consortium (2017). Controlling bias and inflation in epigenome- and transcriptome-wide association studies using the empirical null distribution. Genome Biol. *18*, 19.

40. Higgins, J.P.T., and Thompson, S.G. (2002). Quantifying heterogeneity in a meta-analysis. Stat. Med. *21*, 1539–1558.

41. Hedges, L.V., and Vevea, J.L. (1998). Fixed- and random-effects models in meta-analysis. Psychol. Methods *3*, 486–504.

42. Lee, C.H., Eskin, E., and Han, B. (2017). Increasing the power of meta-analysis of genome-wide association studies to detect heterogeneous effects. In Bioinformatics (Oxford University Press), pp. i379–i388.

43. Shi, J., and Lee, S. (2016). A novel random effect model for GWAS meta-analysis and its application to trans-ethnic meta-analysis. Biometrics *72*, 945–954.

44. Li, Z., Zhao, W., Shang, L., Mosley, T.H., Kardia, S.L.R., Smith, J.A., and Zhou, X. (2022). METRO: multi-ancestry transcriptome-wide association studies for powerful gene-trait association detection. Am. J. Hum. Genet. *109*, 783–801.

45. Baharian, S., Barakatt, M., Gignoux, C.R., Shringarpure, S., Errington, J., Blot, W.J., Bustamante, C.D., Kenny, E.E., Williams, S.M., Aldrich, M.C., and Gravel, S. (2016). The great migration and african-American genomic diversity. PLoS Genet. *12*, e1006059.

46. Zhong, Y., Perera, M.A., and Gamazon, E.R. (2019). On using local ancestry to characterize the genetic architecture of human traits: genetic regulation of gene expression in multiethnic or admixed populations. Am. J. Hum. Genet. *104*, 1097–1115.

47. Mancuso, N., Freund, M.K., Johnson, R., Shi, H., Kichaev, G., Gusev, A., and Pasaniuc, B. (2019). Probabilistic fine-mapping of transcriptome-wide association studies. Nat. Genet. *51*, 675–682.

48. Lu, Z., Gopalan, S., Yuan, D., Conti, D.V., Pasaniuc, B., Gusev, A., and Mancuso, N. (2022). Multi-ancestry fine-mapping improves precision to identify causal genes in transcriptome-wide association studies. Am. J. Hum. Genet. *109*, 1388–1404.

49. Bhattacharya, A., Freedman, A.N., Avula, V., Harris, R., Liu, W., Pan, C., Lusis, A.J., Joseph, R.M., Smeester, L., Hartwell, H.J., et al. (2022). Placental genomics mediates genetic associations with complex health traits and disease. Nat. Commun. *13*, 706.

50. Yang, F., Wang, J., and GTEx Consortium; and Chen, L.S. (2017). Identifying cis-mediators for trans-eQTLs across many human tissues using genomic mediation analysis. Genome Res. *27*, 1859–1871.

51. Veturi, Y., and Ritchie, M.D. (2018). How powerful are summary-based methods for identifying expression-trait associations under different genetic architectures? In Pacific Symposium on Biocomputing (World Scientific Publishing Co. Pte Ltd), pp. 228–239.

52. Barfield, R., Feng, H., Gusev, A., Wu, L., Zheng, W., Pasaniuc, B., and Kraft, P. (2018). Transcriptome-wide association studies accounting for colocalization using Egger regression. Genet. Epidemiol. *42*, 418–433.

53. Yuan, Z., Zhu, H., Zeng, P., Yang, S., Sun, S., Yang, C., Liu, J., and Zhou, X. (2020). Testing and controlling for horizontal pleiotropy with probabilistic Mendelian randomization in transcriptome-wide association studies. Nat. Commun. *11*, 3861–3914.

54. Zhu, A., Matoba, N., Wilson, E.P., Tapia, A.L., Li, Y., Ibrahim, J.G., Stein, J.L., and Love, M.I. (2021). MRLocus: identifying causal genes mediating a trait through Bayesian estimation of allelic heterogeneity. PLoS Genet. *17*, e1009455.

55. Wei, W.Q., Bastarache, L.A., Carroll, R.J., Marlo, J.E., Osterman, T.J., Gamazon, E.R., Cox, N.J., Roden, D.M., and Denny, J.C. (2017). Evaluating phecodes, clinical classification software, and ICD-9-CM codes for phenome-wide association studies in the electronic health record. PLoS One *12*, e0175508.

56. Pathak, G.A., Singh, K., Miller-Fleming, T.W., Wendt, F., Ehsan, N., Hou, K., Johnson, R., Lu, Z., Gopalan, S., Dimbou, L.Y., et al. (2020). Integrative analyses identify susceptibility genes underlying COVID-19 hospitalization. Preprint at medRxiv. https://doi.org/10.1101/2020.12.07.20245308.

57. Unlu, G., Gamazon, E.R., Qi, X., Levic, D.S., Bastarache, L., Denny, J.C., Roden, D.M., Mayzus, I., Breyer, M., Zhong, X., et al. (2019). GRIK5 genetically regulated expression associated with eye and vascular phenomes: discovery through iteration among biobanks, electronic health records, and zebrafish. Am. J. Hum. Genet. *104*, 503–519.

58. Unlu, G., Qi, X., Gamazon, E.R., Melville, D.B., Patel, N., Rushing, A.R., Hashem, M., Al-Faifi, A., Chen, R., Li, B., et al. (2020). Phenome-based approach identifies RIC1 -linked Mendelian syndrome through zebrafish models, biobank associations and clinical studies. Nat. Med. *26*, 98–109.

59. Veturi, Y., Lucas, A., Bradford, Y., Hui, D., Dudek, S., Theusch, E., Verma, A., Miller, J.E., Kullo, I., Hakonarson, H., et al. (2021). A unified framework identifies new links between plasma lipids and diseases from electronic medical records across large-scale cohorts. Nat. Genet. *53*, 972–981.

60. Johansson, Å., Rask-Andersen, M., Karlsson, T., and Ek, W.E. (2019). Genome-wide association analysis of 350 000 Caucasians from the UK Biobank identifies novel loci for asthma, hay fever and eczema. Hum. Mol. Genet. *28*, 4022–4041.

61. Portelli, M.A., Dijk, F.N., Ketelaar, M.E., Shrine, N., Hankinson, J., Bhaker, S., Grotenboer, N.S., Obeidat, M., Henry, A.P., Billington, C.K., et al. (2020). Phenotypic and functional translation of IL1RL1 locus polymorphisms in lung tissue and asthmatic airway epithelium. JCI Insight *5*, 132446.

62. Reijmerink, N.E., Postma, D.S., Bruinenberg, M., Nolte, I.M., Meyers, D.A., Bleecker, E.R., and Koppelman, G.H. (2008). Association of IL1RL1, IL18R1, and IL18RAP gene cluster polymorphisms with asthma and atopy. J. Allergy Clin. Immunol. *122*, 651–654.e8.

63. Reijmerink, N.E., Postma, D.S., and Koppelman, G.H. (2010). The candidate gene approach in asthma: what happens with the neighbours? Eur. J. Hum. Genet. *18*, 17.

64. Zhu, Z., Guo, Y., Shi, H., Liu, C.L., Panganiban, R.A., Chung, W., O'Connor, L.J., Himes, B.E., Gazal, S., Hasegawa, K., et al. (2020). Shared genetic and experimental links between obesity-related traits and asthma subtypes in UK Biobank. J. Allergy Clin. Immunol. *145*, 537–549.

65. Hebbring, S.J. (2014). The challenges, advantages and future of phenome-wide association studies. Immunology *141*, 157–165.

66. Korthauer, K., Kimes, P.K., Duvallet, C., Reyes, A., Subramanian, A., Teng, M., Shukla, C., Alm, E.J., and Hicks, S.C. (2019). A practical guide to methods controlling false discoveries in computational biology. Genome Biol. *20*, 118–121.

67. Stevens, J.R., Al Masud, A., and Suyundikov, A. (2017). A comparison of multiple testing adjustment methods with block-correlation positivelydependent tests. PLoS One *12*, e0176124.
68. McGuirl, M.R., Smith, S.P., Sandstede, B., and Ramachandran, S. (2020). Detecting shared genetic architecture among multiple phenotypes by hierarchical clustering of gene-level association statistics. Genetics *215*, 511–529.
69. Hu, Y., Li, M., Lu, Q., Weng, H., Wang, J., Zekavat, S.M., Yu, Z., Li, B., Gu, J., Muchnik, S., et al. (2019). A statistical framework for cross-tissue transcriptome-wide association analysis. Nat. Genet. *51*, 568–576.
70. Thompson, M., Gordon, M.G., Lu, A., Tandon, A., Halperin, E., Gusev, A., Ye, C.J., Balliu, B., and Zaitlen, N. (2021). Multi-context genetic modeling of transcriptional regulation resolves novel disease loci. Preprint at bioRxiv. https://doi.org/10.1101/2021.09.23.461579.
71. Lu, A., Thompson, M., Gordon, M.G., Dahl, A., Ye, C.J., Zaitlen, N., and Balliu, B. (2021). Fast and Powerful Statistical Method for Context-specific QTL mapping in multi-context genomic studies. Preprint at bioRxiv. https://doi.org/10.1101/2021.06.17.448889.
72. Liu, W., Deng, W., Chen, M., Dong, Z., Zhu, B., Yu, Z., Sauler, M., Wain, L.V., Cho, M.H., Kaminski, N., et al. (2021). A statistical framework to identify cell types whose genetically regulated proportions are associated with complex diseases. Preprint at medRxiv. https://doi.org/10.1101/2021.02.25.21252462.
73. Burkhardt, D.B., Stanley, J.S., Tong, A., Perdigoto, A.L., Gigante, S.A., Herold, K.C., Wolf, G., Giraldez, A.J., van Dijk, D., and Krishnaswamy, S. (2021). Quantifying the effect of experimental perturbations at single-cell resolution. Nat. Biotechnol. *39*, 619–629.
74. Verma, A., and Engelhardt, B.E. (2020). A robust nonlinear low-dimensional manifold for single cell RNA-seq data. BMC Bioinf. *21*, 324–415.
75. Baca, S., Singler, C., Zacharia, S., Seo, J.-H., Morova, T., Hach, F., Ding, Y., Schwarz, T., Flora Huang, C.-C., Kalita, C., et al. (2021). Genetic determinants of chromatin reveal prostate cancer risk mediated by context-dependent gene regulation. Preprint at bioRxiv. https://doi.org/10.1101/2021.05.10.443466.
76. Zhao, H., Rasheed, H., Nøst, T.H., Cho, Y., Liu, Y., Bhatta, L., Bhattacharya, A., Initiative, G.B.M., Hemani, G., Smith, G.D., et al. (2022). Proteome-wide mendelian randomization in global biobank meta-analysis reveals multi-ancestry drug targets for common diseases. Preprint at medRxiv. https://doi.org/10.1101/2022.01.09.21268473.
77. Bhattacharya, A., and Hirbo, J.B. (2022). bhattacharya-a-bt/gbmi_twas.
78. Bycroft, C., Freeman, C., Petkova, D., Band, G., Elliott, L.T., Sharp, K., Motyer, A., Vukcevic, D., Delaneau, O., O'Connell, J., et al. (2018). The UK Biobank resource with deep phenotyping and genomic data. Nature *562*, 203–209.

CellPress
OPEN ACCESS

STAR★METHODS

KEY RESOURCES TABLE

REAGENT or RESOURCE	SOURCE	IDENTIFIER
Deposited data		
GBMI GWAS summary statistics	Zhou et al. 2022	https://www.globalbiobankmeta.org/resources
The Geno-type-Tissue Expression Project v8	Aguet et al. 2020	dbGaP Study Accession: phs000424.v8.p2
1000Genomes Project, Phase 3	Auton et al. 2015	ftp://ftp.1000genomes.ebi.ac.uk/vol1/ftp/data_collections/1000_genomes_project/data
Software and algorithms		
MR-JTI	Zhou et al. 2020	https://github.com/gamazonlab/MR-JTI
MOSTWAS	Bhattacharya et al. 2021	https://github.com/bhattacharya-a-bt/MOSTWAS
Glmnet	Friedman et al. 2010	https://cran.r-project.org/web/packages/glmnet/
FOCUS	Mancuso et al. 2019	https://github.com/bogdanlab/focus
Scripts for GBMI TWAS	Bhattacharya et al. 2022	https://github.com/bhattacharya-a-bt/gbmi_twas

RESOURCE AVAILABILITY

Lead contact

Further information and requests for data availability and code should be directed to and will be fulfilled by the lead contact, Arjun Bhattacharya (abtbhatt@ucla.edu).

Materials availability

No materials were generated in this study.

Data and code availability

The all-biobank and ancestry-specific GWAS summary statistics are publicly available for downloading at https://www.globalbiobankmeta.org/resources and browsed at the PheWeb Browser http://results.globalbiobankmeta.org/. 1000Genomes Phase 3 data can be accessed at ftp://ftp.1000genomes.ebi.ac.uk/vol1/ftp/data_collections/1000_genomes_project/data. MOSTWAS can be accessed from https://github.com/bhattacharya-a-bt/MOSTWAS, and JTI can be accessed from https://github.com/gamazonlab/MR-JTI. Sample scripts for this manuscript are available at https://github.com/bhattacharya-a-bt/gbmi_twas.[77]

EXPERIMENTAL MODEL AND SUBJECT DETAILS

No experimental models were employed here. We use data from the Genotype-Tissue Expression Project (GTEx)[28] version 8 for 5 tissues: subcutaneous adipose (N = 492 EUR, N = 71 AFR), tibial artery (N = 489 EUR, N = 76 AFR), skeletal muscle (N = 602 EUR, N = 86 AFR), sun exposed lower leg skin (N = 518 EUR, N = 73 AFR), and whole blood (N = 574 EUR, N = 80 AFR). Ancestry groups were specified by GTEx. Sample sizes and acquisition details are provided in detail in Zhou et al.[3]

METHOD DETAILS

We first outline the steps of the TWAS pipeline employed for phenotype available for analysis in the GBMI. Then, we provide details for the analyses presented in Results.

The GBMI TWAS pipeline

Training expression models from genetics

Tissue-specific expression models trained with reference data from the Genotype-Tissue Expression Project (GTEx) v8[28] are built using two methods: (1) Joint-Tissue Imputation (JTI), which leverages shared genetic *cis*-regulation across tissues,[26] and (2) MOSTWAS, which prioritizes tissue-specific distal-SNPs through rigorous mediation analysis to account for additional expression heritability.[17] Genes with significantly positive expression heritability (nominal $P < 0.01$) and five-fold cross-validation (CV) adjusted

$R^2 \geq 0.01$ with $P < 0.05$ are considered for TWAS. Ancestry-specific models are trained, excluding SNPs with MAF <0.01 and deviated from Hardy-Weinberg at $P < 10^{-5}$across all 838 GTEx samples. These filtering steps led to a total of 6,106,016 SNPs. We acknowledge that removing SNPs that deviate from Hardy-Weinberg may lead to removal of some causal eQTL variants, but often times these SNPs are prone to genotyping errors. In particular, we assessed a less conservative p-value cutoff for deviation from Hardy-Weinberg ($p < 10^{-6}$) as in previous TWAS analyses[13,14,23]; this cutoff only included an additional 47,265 SNPs. A comprehensive analysis of the effects of estimated deviation from Hardy-Weinberg on eQTL estimation and TWAS predictive model training will be illuminating. The first iteration of the GBMI TWAS pipeline focuses on EUR-ancestry models due to larger sample sizes. However, as sample sizes for reference eQTL data for other ancestry groups increase, this pipeline will include gene expression models that are specific for these currently underrepresented ancestry groups. In addition, models from other data sources using other methods can be incorporated in subsequent steps.

Hypothesis tests for TWAS

To test for an association between tissue-specific GReX of a gene and a trait of interest, GWAS summary statistics are integrated with these expression models. For the EUR-specific TWAS, we use EUR-specific meta-analyzed GWAS summary statistics across all biobanks. JTI and MOSTWAS use two different approaches to test for a GTA. For MR-JTI, the posterior predictive distribution of GReX is estimated, and multiple-instrumental-variable causal inference is used to estimate the GTA, controlling for overall heterogeneity.[26] For MOSTWAS, a weighted burden test is constructed, as in FUSION.[14,17] Both of these methods require a LD reference panel; the GTEx LD matrix is used as a reference. Taken together, these methods provide effect sizes, standard errors, Z-scores (effect sizes standardized by standard error), and p-values for GTAs. A GTA is transcriptome-wide significant using a Bonferroni correction across all tests run. The number of tests run is equal to the sum of the number of significant gene models across all tissues.

Follow-up tests and analyses are then run to provide context to the TWAS GTAs. A permutation test is run by shuffling the SNP-gene weights 1,000 times and determining the TWAS Z score at each permutation, generating a null distribution. The original TWAS Z-score is compared to this null distribution to generate a permutation p-value; Benjamini-Hochberg FDR correction is used to account for multiple testing burden here. This test examines whether the SNP-gene relationship provides more information than just the SNP-trait association. Next, for MOSTWAS, the distal-SNPs added-last test is run to measure the association from distal-SNPs in the expression models, conditional on the association from local-SNPs.[17] This test prioritizes sets of mediating molecular features for the SNP-gene relationship with significant effects on the trait. Lastly, for genes whose models are built using SNPs from overlapping genomic regions, probabilistic fine-mapping via FOCUS (default parameters and priors) is employed to determine a 90% credible set of genes that explain the gene-level association signal at the locus.[47] FOCUS also outputs posterior inclusion probabilities for each gene in the 90% credible set.

Analysis of ancestry-specific and -unaware models

To show the utility of ancestry-specific models, we train EUR- and AFR-specific models using elastic net regression for 5 tissues with more than 70 samples from AFR ancestry patients. To balance sample sizes in the imputation sample, we down-sampled the EUR ancestry imputation sample to match the AFR imputation sample. We consider only genes with positive expression heritability in both EUR and AFR training samples. We also build ancestry-unaware models, where genotypes for EUR and AFR samples are pooled together in the training sample. We calculate predictive performance in aligned and misaligned imputation samples based on ancestry; the aligned imputation sample is one with ancestry that predominantly matches the ancestry of the training sample. Predictive performance is measured with adjusted R^2 to account for sample size, using an appropriate linear model between predicted and observed expression. For imputation samples that are used in training (aligned imputation panel), we use 5-fold CV when measuring predictive performance. We also conducted a down-sampling analysis, where we randomly selected a subset of the EUR samples to match the AFR sample size; in this down-sample, we performed the same comparison of predictive performance. Lastly, when imputing into AFR and EUR samples using the ancestry-unaware models, we only cross-validate over the AFR or EUR samples, respectively. For example, the training set in a fold when imputed into AFR samples includes all EUR samples and 80% of the AFR samples.

QUANTIFICATION AND STATISTICAL ANALYSIS

Strategies for meta-analysis

We compared 5 different meta-analytic strategies empirically: meta-analyzing across ancestry-specific, per-biobank GWAS summary statistics using (1) inverse-variance weighting (IVW) and (2) sample-size weighting (SSW), meta-analyzing across ancestry-specific meta-analyzed GWAS summary statistics using (3) IVW and (4) SSW, and (5) TWAS using ancestry-unaware models into meta-analyzed GWAS summary statistics across EUR and AFR ancestry groups. First, we consider three different sets of GWAS summary statistics: biobank- and ancestry-specific summary statistics, ancestry-specific summary statistics meta-analyzed across all biobanks, and summary statistics meta-analyzed across biobanks and ancestry groups. In two former settings, for biobank *i* and a given gene, we generate $\beta_{TWAS,i}$, the TWAS effect size, and $SE_{TWAS,i}$, the corresponding standard error. Given *B* different biobanks, the IVW TWAS Z-score, $Z_{TWAS,\ IVW}$, is calculated as:

$$Z_{TWAS,IVW} = \frac{\left(\frac{\sum_{i=1}^{B} \beta_{TWAS,i} \Big/ SE_{TWAS,i}}{\sum_{i=1}^{B} SE_{TWAS,i}^{-1}}\right)}{\left(\sum_{i=1}^{B} SE_{TWAS,i}\right)^{1/2}}.$$

With $Z_{TWAS,i} = \beta_{TWAS,i}/SE_{TWAS,i}$ and N_i as the effective sample size of the i th biobank (or pooled effective sample size across all ancestry-specific biobank summary statistics), the SSW TWAS Z-score, $Z_{TWAS,SSW}$, is calculated as:

$$Z_{TWAS,SSW} = \frac{\sum_{i=1}^{B} N_i Z_{TWAS,i}}{\left(\sum_{i=1}^{B} N_i^2\right)^{1/2}}.$$

Here, we define the effective sample size as $N_i = 4/\left(\frac{1}{N_{cases}} + \frac{1}{N_{controls}}\right)$.

For the ancestry-unaware TWAS, we use ancestry-unaware elastic net regression models and integrate with GWAS summary statistics meta-analyzed across all ancestry groups and biobanks.

GReX-level phenome-wide association studies (GReX-PheWAS)

Transcriptome-wide significant genes are further prioritized by performing GReX-PheWAS to categorize associations across a broad spectrum of phenotypes. Using UKBB summary statistics from European ancestry patients,[78] we tested for GTAs for 731 phenotypes grouped into 9 categories: dermatologic, digestive, endocrine/metabolic, genitourinary, hematopoietic, musculoskeletal, neoplasms, neurological, and respiratory. Here, we illustrate GReX-PheWAS using three genes from the European-specific TWAS for asthma risk using lung tissue expression: *TAF7* (MOSTWAS model), *IL18RAP* (JTI model), and *TMEM258* (JTI model). A phenome-wide significant association was defined via Bonferroni correction ($P < \frac{0.05}{3 \times 731} = 2.28 \times 10^{-5}$).

Cell Genomics, Volume *2*

Supplemental information

Best practices for multi-ancestry, meta-analytic transcriptome-wide association studies: Lessons from the Global Biobank Meta-analysis Initiative

Arjun Bhattacharya, Jibril B. Hirbo, Dan Zhou, Wei Zhou, Jie Zheng, Masahiro Kanai, the Global Biobank Meta-analysis Initiative, Bogdan Pasaniuc, Eric R. Gamazon, and Nancy J. Cox

SUPPLEMENTAL INFORMATION

Best practices of multi-ancestry, meta-analytic transcriptome-wide associations: lessons from the Global Biobank Meta-Initiative

SUPPLEMENTAL FIGURES

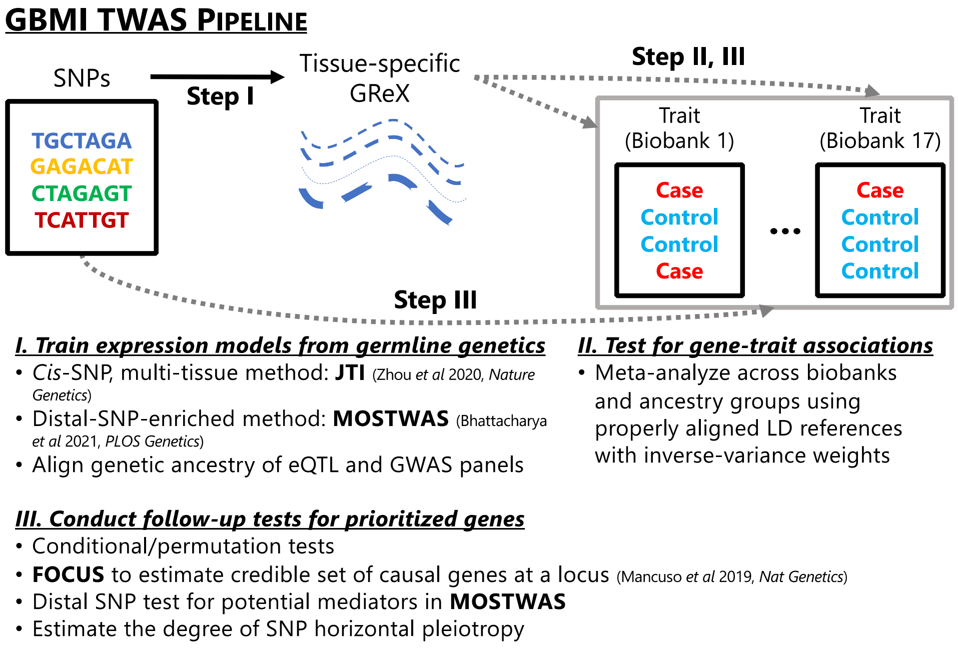

Figure S1: *An overview of the GBMI TWAS pipeline*. (Step 1) JTI and MOSTWAS for model training, (Step 2) inverse-variance weighted meta-analysis using per-biobank, per-ancestry group TWAS summary statistics, and (Step 3) various follow-up tests, including conditional or permutation tests, distal-SNPs added last test, probabilistic fine-mapping using FOCUS, and tests for SNP horizontal pleiotropy. Dotted lines represent associations that are tested in the TWAS pipeline, while the solid lines represent a link built through predictive modeling. Related to Figure 1.

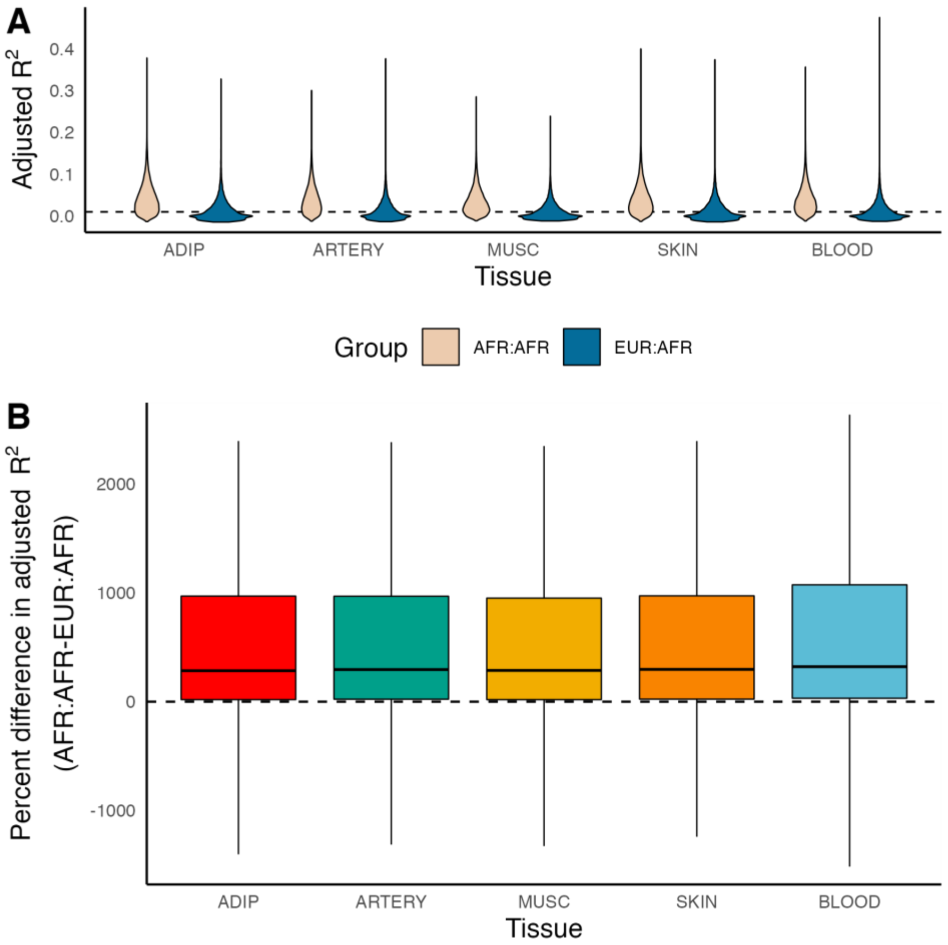

Figure S2: (A) Distribution of adjusted R^2 across 5 tissues using ancestry-aligned (AFR:AFR) models and ancestry-misaligned (EUR:AFR) models. (B) Boxplot of percent difference in adjusted R^2 using ancestry-aligned (AFR:AFR) models versus ancestry-misaligned (EUR:AFR) models. Related to Figure 2.

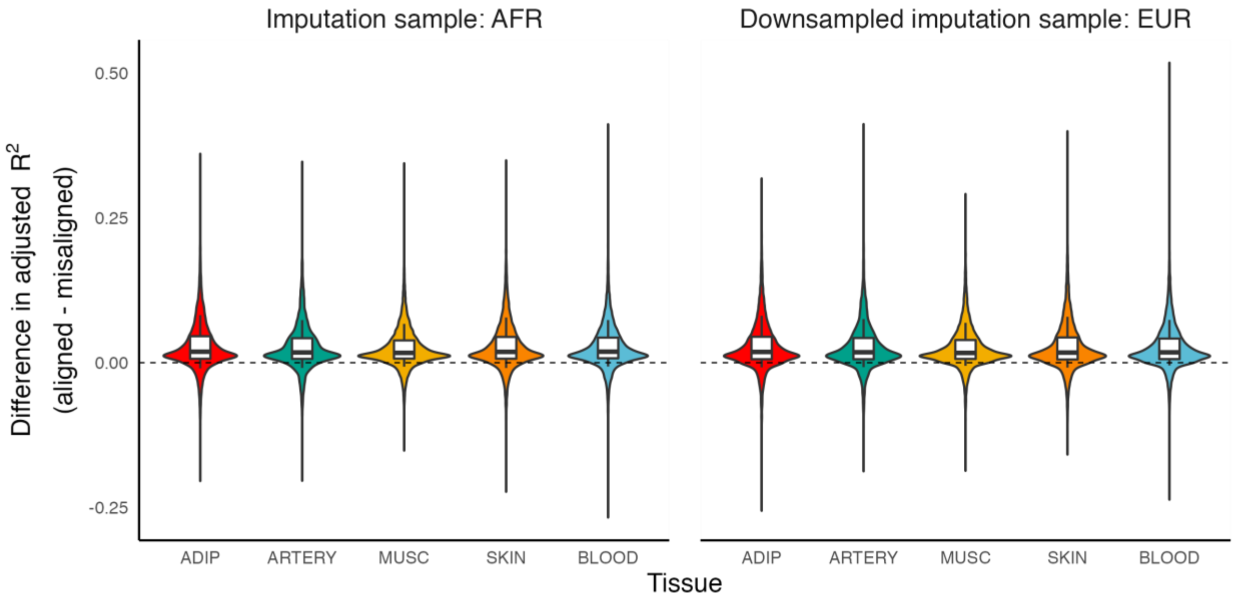

Figure S3: *Difference of predictive performance of expression models in aligned versus misaligned imputation samples across AFR (left) and EUR (right) ancestry in the imputation sample.* Here, we down-sample the EUR imputation sample to match the sample size of the AFR imputation sample. Related to Figure 2.

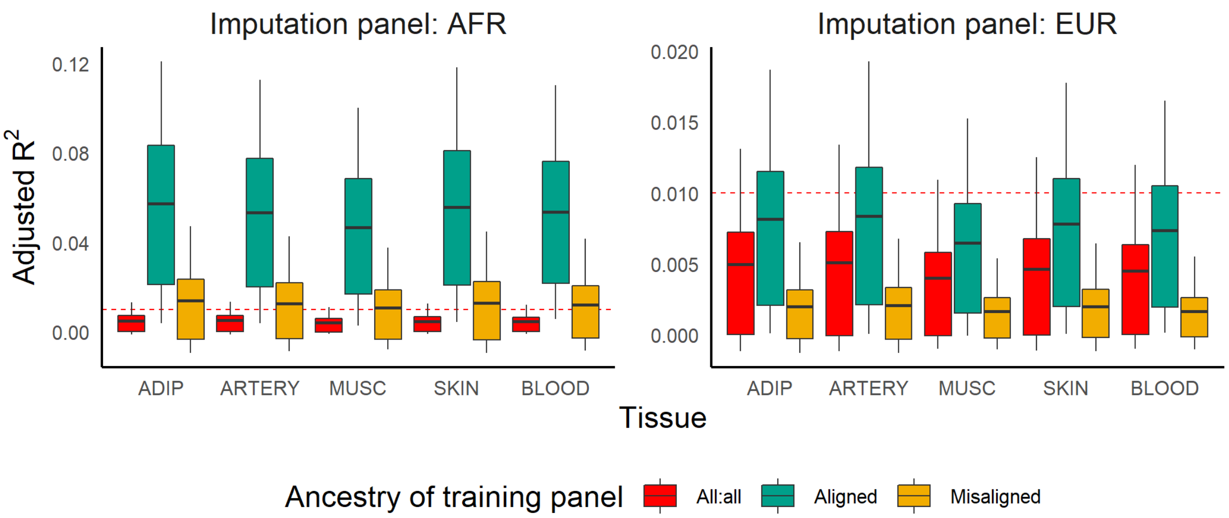

Figure S4: *Predictive performance of expression models in aligned and misaligned imputation samples*. Related to Figure 2.

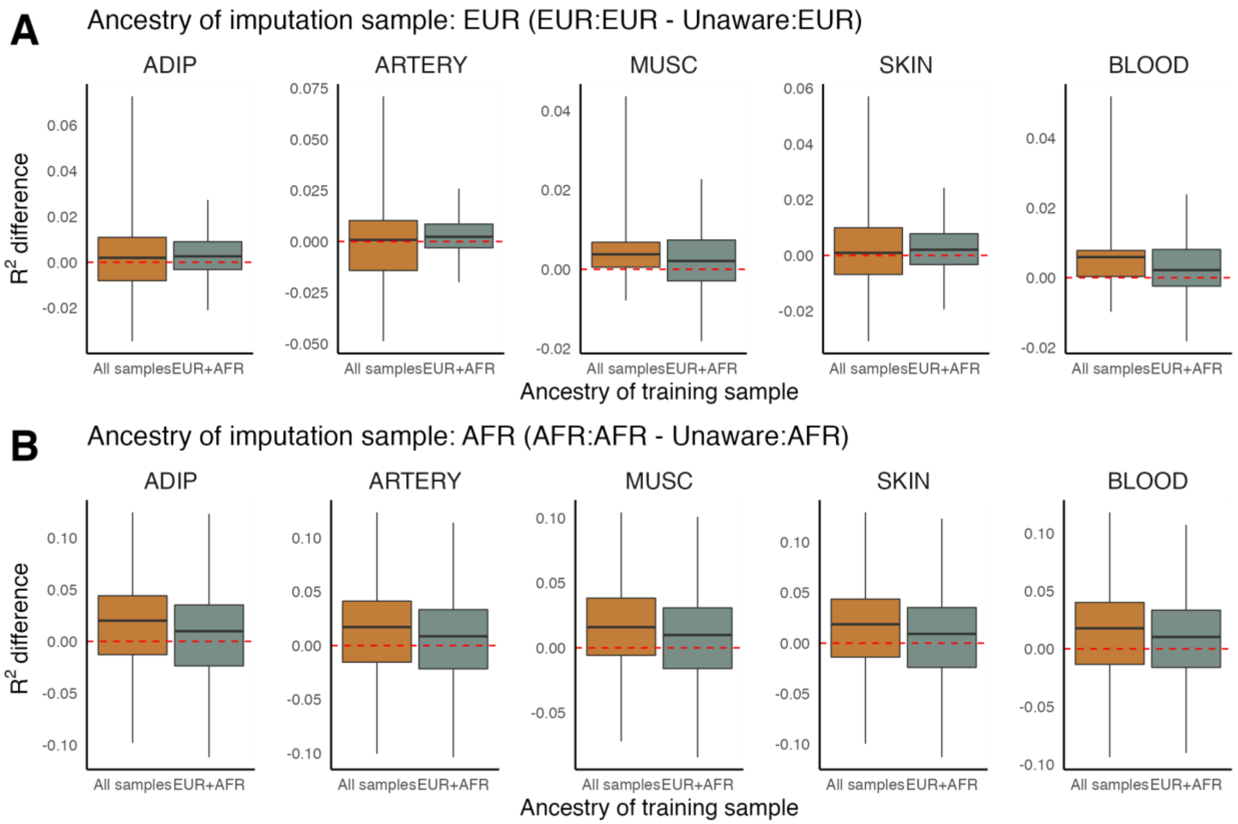

Figure S5: *Predictive performance of ancestry-unaware expression models compared to ancestry-specific models across 5 tissues.* Boxplot of difference in predictive performance in EUR **(A)** and AFR **(B)** samples between ancestry-aligned models and ancestry-unaware models. We consider (1) individuals of all ancestry in the training sample of the ancestry-unaware model (gold) or only EUR and AFR individuals in the training sample (grey). The red line indicates a difference of 0. Related to Figure 2.

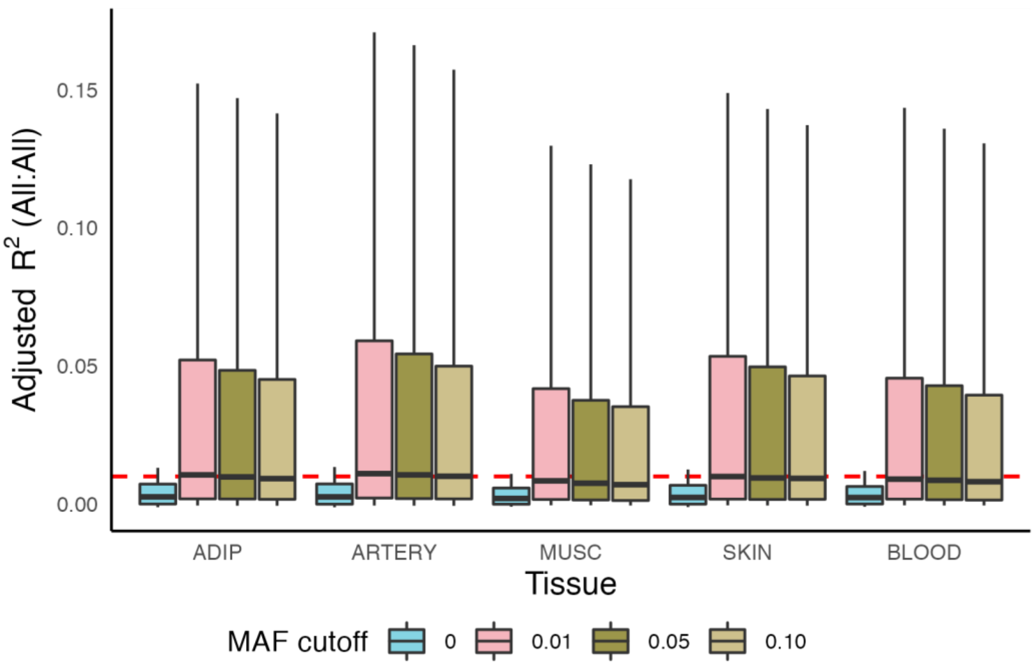

Figure S6: *Predictive performance of ancestry-unaware expression models across minor allele frequency thresholds*. Related to Figure 2.

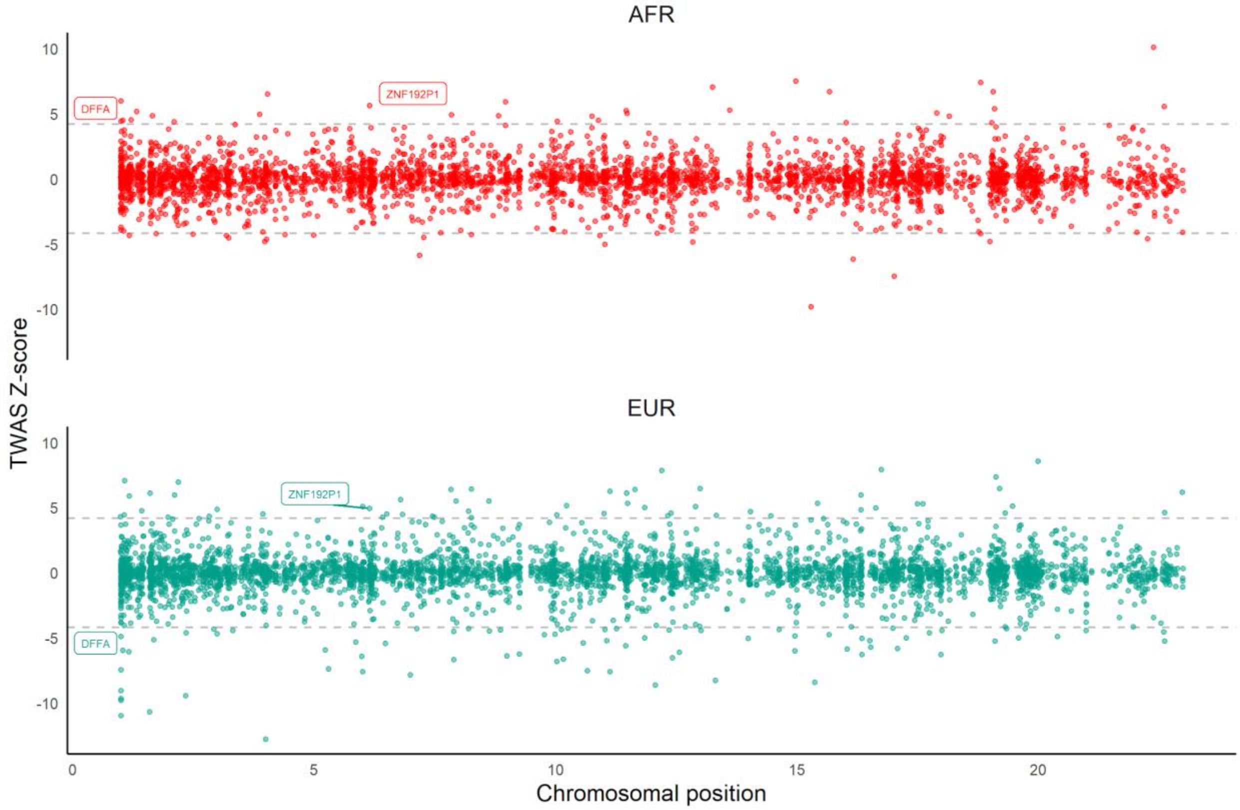

Figure S7: *TWAS Miami plots across AFR and EUR ancestry groups for asthma using whole blood gene expression models*. Related to Figure 3.

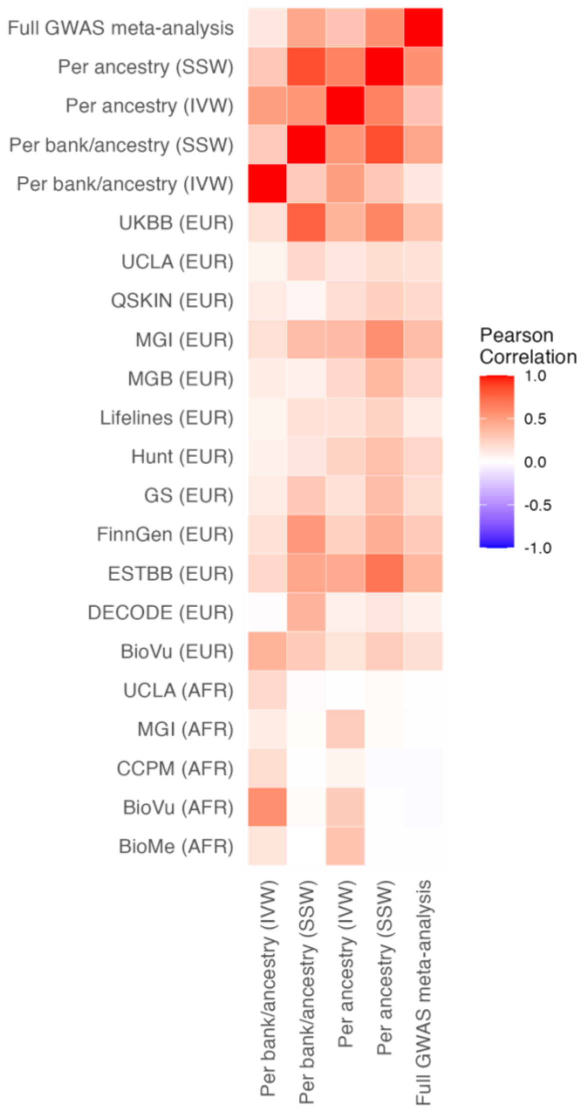

Figure S8: *Correlation of TWAS Z-scores across ancestry-specific, individual biobank GWAS cohorts and 5 meta-analytic strategies.* Related to Figure 3.

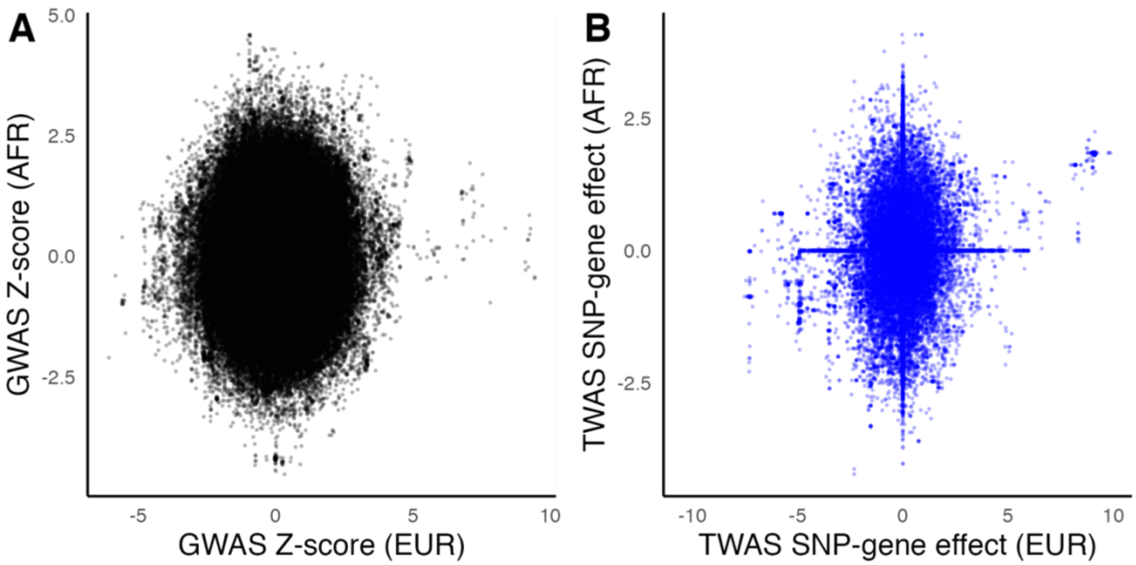

Figure S9: Correlation of meta-analyzed GWAS Z-scores (A) and TWAS SNP-gene effects (B) across EUR (X-axis) and AFR (Y-axis) ancestry groups. Related to Figure 3.

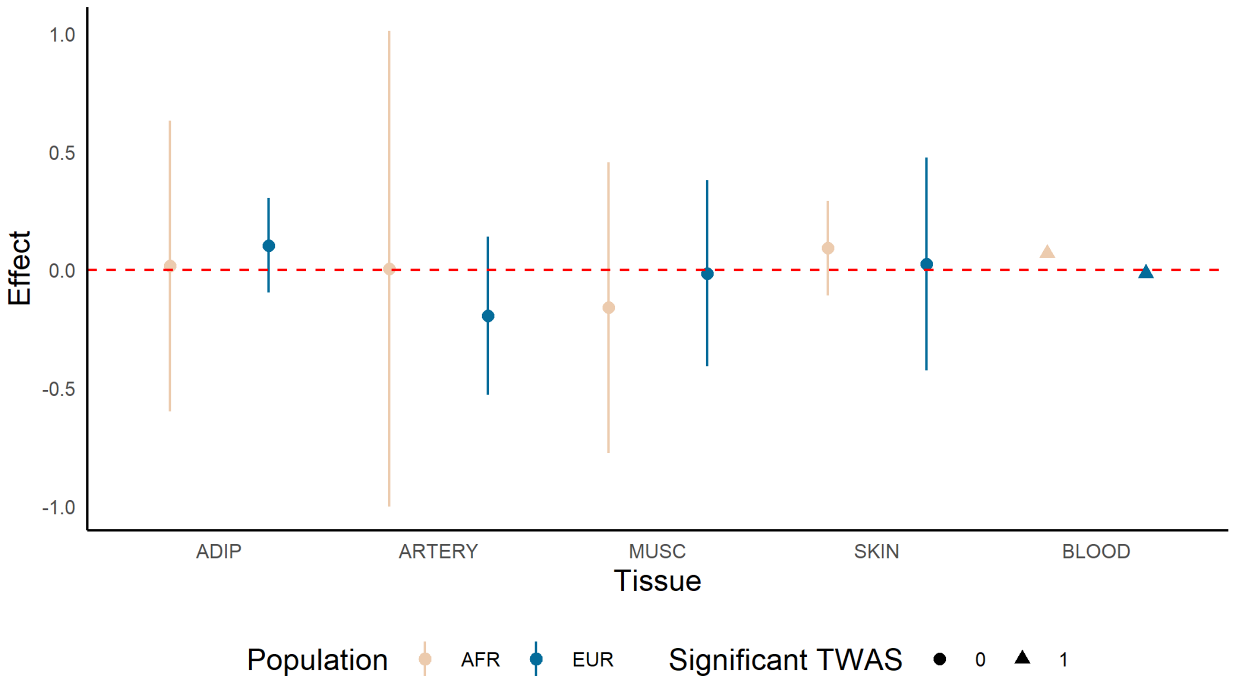

Figure S10: *TWAS associations across EUR and AFR ancestry groups for DFFA across 5 tissues.* The effect size is given with the point (triangle if association is transcriptome-wide significant) with a 95% confidence interval provided. Related to Figure 3.

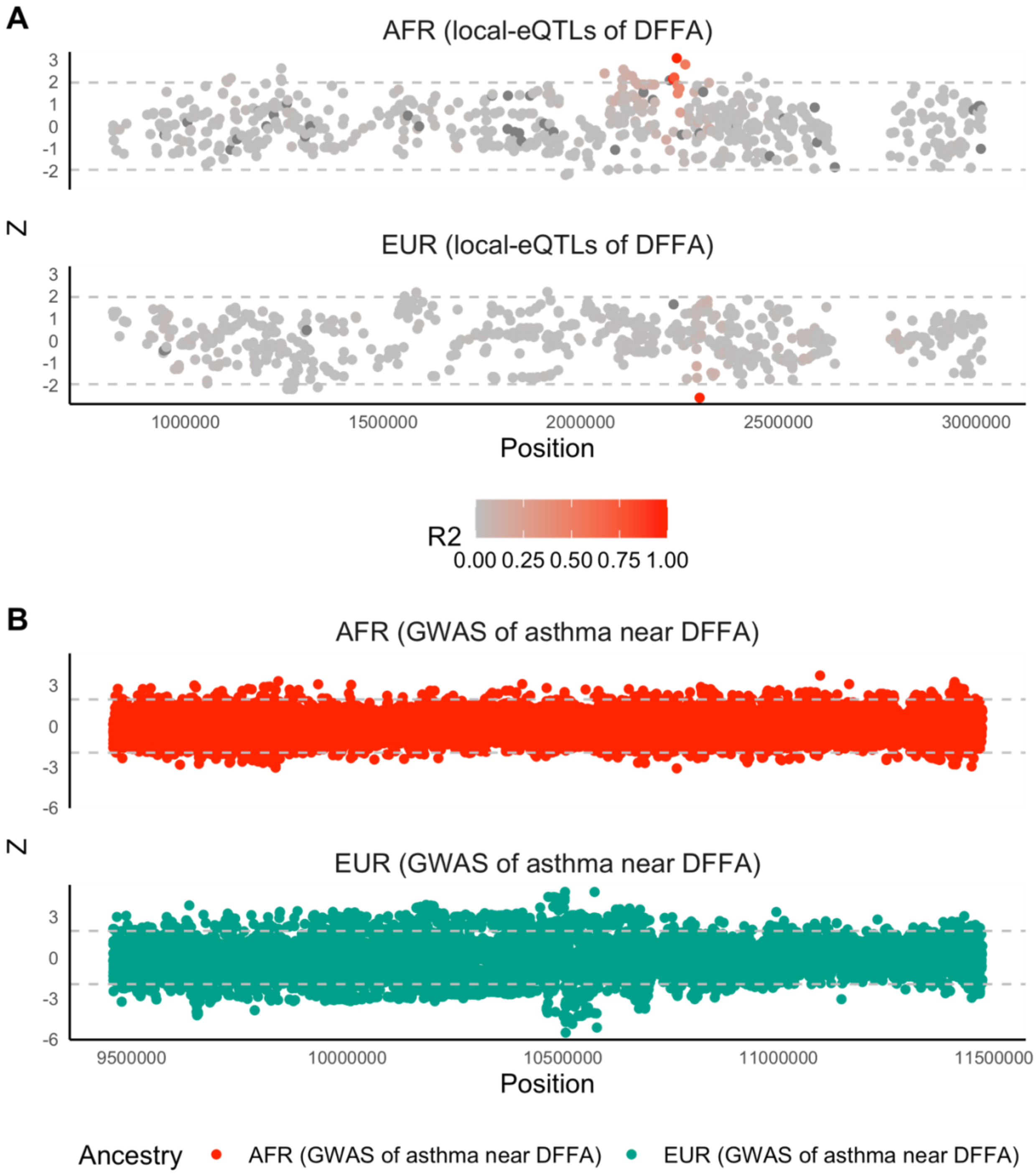

Figure S11: *Miami plots of DFFA local-eQTLs and GWAS signal for SNPs around DFFA.* In (A), color shows linkage disequilibrium R^2 to lead eQTL SNP. Grey line shows a nominal P-value cutoff of 0.05 ($|Z| = 1.96$). Related to Figure 3.

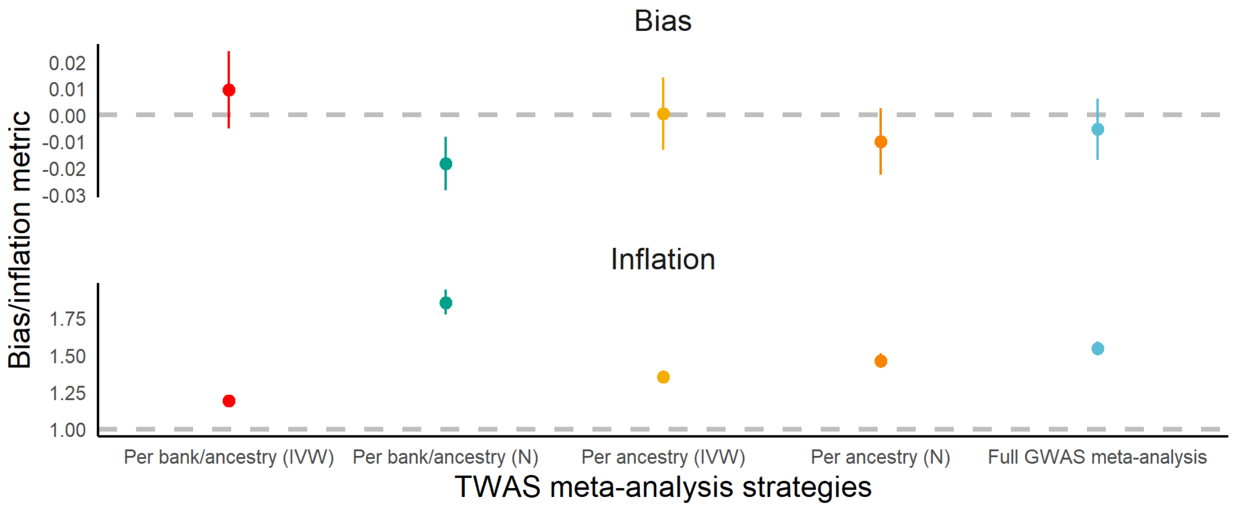

Figure S12: *Empirical Bayes estimates of bias and inflation in TWAS Z-scores across meta-analysis strategies.* Estimates of bias (top) and bottom (inflation) with one standard error width around the estimate are given across meta-analysis strategies. The dotted lines provide a reference for the null (0 for bias and 1 for inflation). Related to Figure 3.

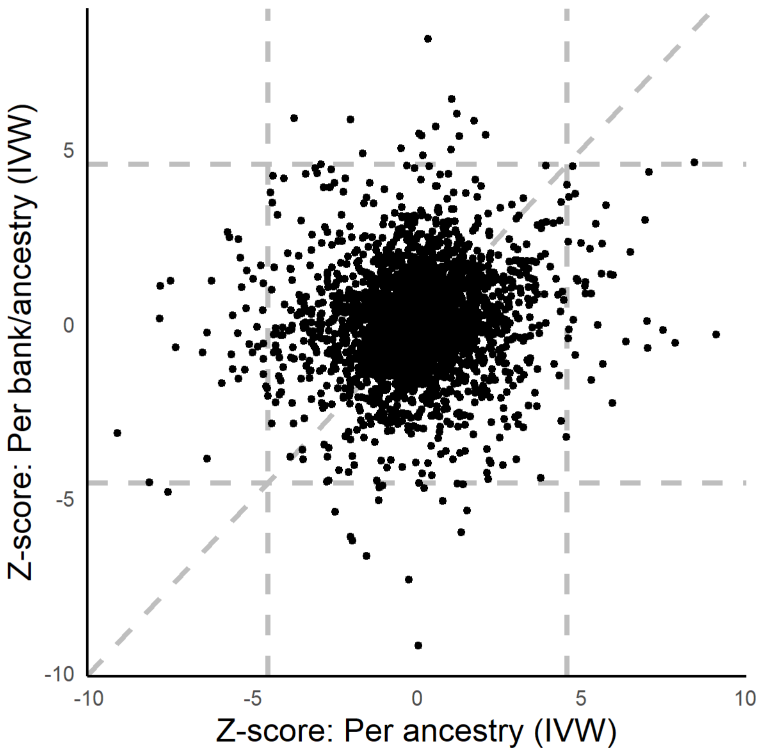

Figure S13: *Comparison of two IVW meta-analyzed Z-scores.* Vertical and horizontal dotted lines give a reference for the Bonferroni-corrected threshold for transcriptome-significance. A diagonal line is provided from reference. Related to Figure 3.

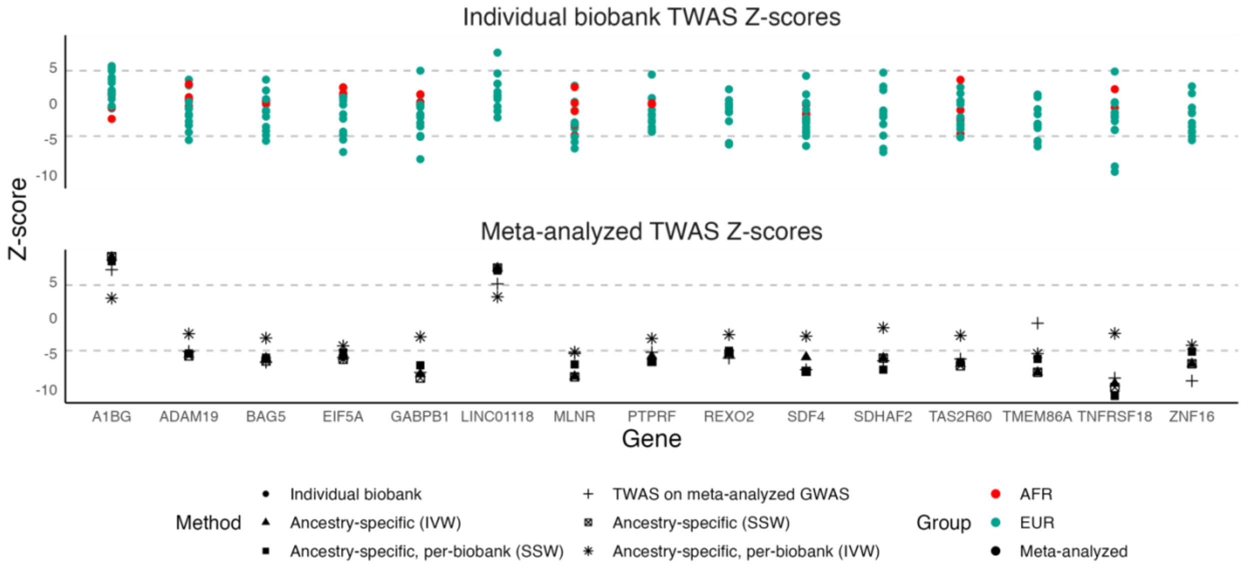

Figure S14: *Comparison of meta-analyzed Z-scores with individual biobank TWAS Z-scores.* Ancestry-specific TWAS Z-scores for individual biobanks are shown in the top panel, colored by ancestry. Meta-analyzed Z-scores are shown in the bottom panel with shapes reflecting the different strategies. Dotted lines provide a reference for transcriptome-wide significance. Related to Figure 3.

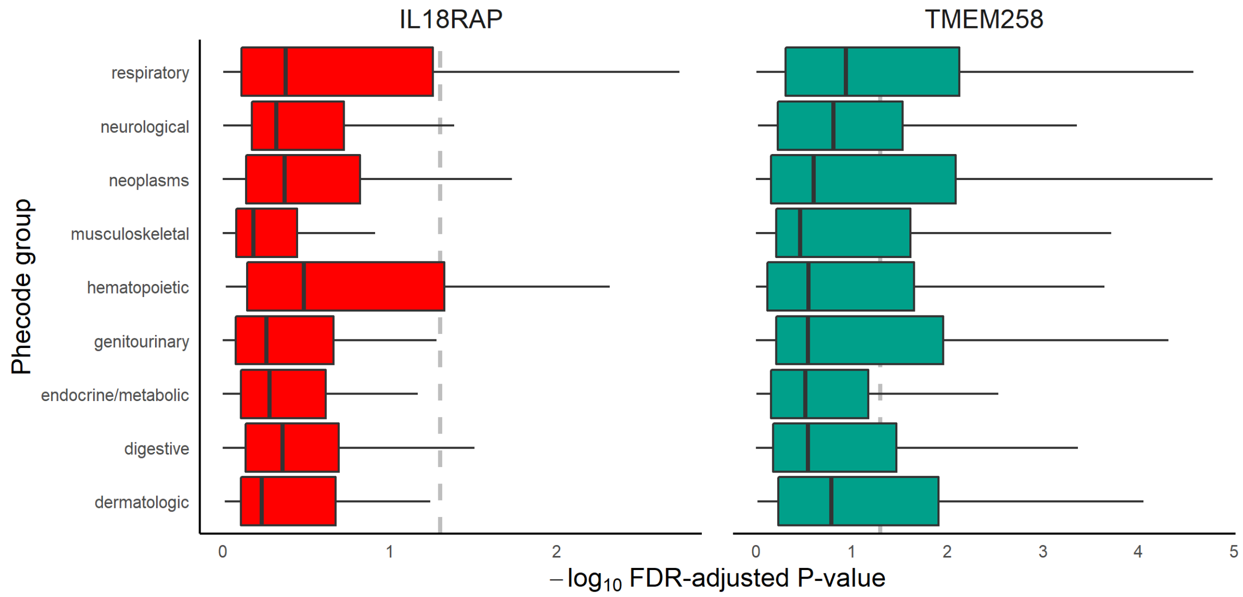

Figure S15: *UKBB GReX-PheWAS associations across 5 representative asthma-associated genes through European-only meta-analytic TWAS, grouped by phecode group.* The horizontal dotted line shows FDR-adjusted P = 0.05. Related to Figure 4.

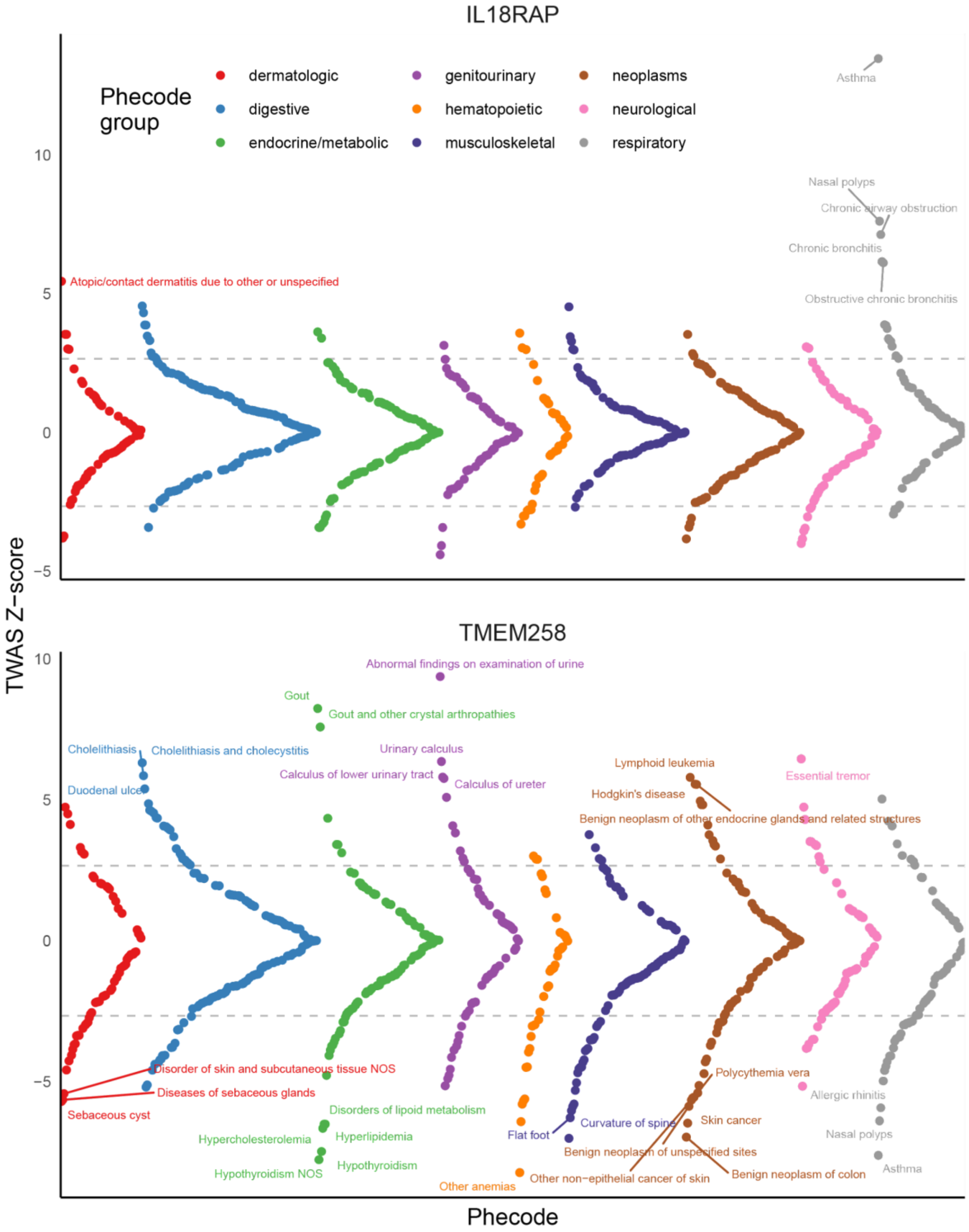

Figure S16: *Miami plots of UKBB GReX-PheWAS associations across 2 genes previously implicated through GWAS and detected in European-only meta-analytic TWAS in GBMI.* Related to Figure 4.

SUPPLEMENTAL TABLES

Table S1: Difference in adjusted R2 between models trained in aligned and misaligned ancestry samples as the ancestry of the imputation sample. Related to Figure 2.

	Imputation sample ancestry: down-sampled EUR					Imputation sample ancestry: AFR				
**Tissue**	**Mean**	**Median**	**25% Quantile**	**75% Quantile**	**Percent > 0**	**Mean**	**Median**	**25% Quantile**	**75% Quantile**	**Percent > 0**
**ADIP**	0.018	0.009	3.77E-04	3.50E-02	73.4	0.018	0.009	0.008	0.036	77.6
**ARTERY**	0.017	0.008	3.00E-04	3.30E-02	73.5	0.016	0.008	0.008	0.033	78.3
**MUSC**	0.015	0.007	5.27E-04	2.90E-02	74.1	0.015	0.008	0.010	0.036	77.4
**SKIN**	0.017	0.007	1.89E-04	3.40E-02	72.5	0.010	0.009	0.006	0.034	78.2
**BLOOD**	0.017	0.008	3.12E-04	3.20E-02	73.7	0.017	0.009	0.008	0.033	79.4

Table S2: R^2 of ancestry-specific models imputed into EUR imputation sample (training:imputation). Related to Figure 2.

	EUR:EUR				AFR:EUR			
**Tissue**	**Mean**	**Median**	**25% Quantile**	**75% Quantile**	**Mean**	**Median**	**25% Quantile**	**75% Quantile**
**ADIP**	0.008	0.006	2.02E-03	1.12E-02	0.002	0.001	-2.64E-04	3.19E-03
**ARTERY**	0.008	0.006	2.05E-03	1.15E-02	0.002	0.001	-3.15E-04	3.33E-03
**MUSC**	0.007	0.005	1.50E-03	9.00E-03	0.002	0.001	-2.29E-04	2.64E-03
**SKIN**	0.008	0.006	1.90E-03	1.10E-02	0.002	0.001	-1.85E-04	3.20E-03
**BLOOD**	0.007	0.005	1.92E-03	1.03E-02	0.002	0.001	-1.22E-04	2.64E-03

Table S3: R^2 of ancestry-specific models imputed into AFR imputation sample (training:imputation). Related to Figure 2.

	AFR:AFR				EUR:AFR			
**Tissue**	**Mean**	**Median**	**25% Quantile**	**75% Quantile**	**Mean**	**Median**	**25% Quantile**	**75% Quantile**
ADIP	0.043	0.035	0.015	0.062	0.014	0.006	-0.003	0.023
ARTERY	0.040	0.033	0.015	0.058	0.013	0.005	-0.003	0.022
MUSC	0.035	0.028	0.012	0.051	0.011	0.004	-0.003	0.019
SKIN	0.042	0.034	0.015	0.060	0.013	0.005	-0.004	0.023
BLOOD	0.040	0.034	0.016	0.057	0.012	0.005	-0.003	0.021

Table S4: Difference in R^2 between ancestry-specific and ancestry-unaware models across MAF. Related to Figure 2.

		AFR:AFR - All:AFR					EUR:EUR - All:EUR				
**Tissue**	**MAF**	**Mean**	**Median**	**25% Quantile**	**75% Quantile**	**Proportion > 0**	**Mean**	**Median**	**25% Quantile**	**75% Quantile**	**Proportion > 0**
**ADIP**	0	0.004	0.003	-0.003	0.009	0.634	0.028	0.024	-0.009	0.066	0.689
**ARTERY**	0	0.003	0.002	-0.003	0.009	0.620	0.026	0.022	-0.010	0.060	0.679
**MUSC**	0	0.004	0.002	-0.003	0.008	0.625	0.025	0.022	-0.014	0.061	0.664
**SKIN**	0	0.003	0.002	-0.003	0.008	0.622	0.023	0.020	-0.009	0.053	0.674
**BLOOD**	0	0.003	0.002	-0.003	0.008	0.635	0.028	0.027	-0.004	0.060	0.724
**ADIP**	0.01	-0.001	-0.001	-0.006	0.004	0.375	0.026	0.028	0.002	0.086	0.750
**ARTERY**	0.01	0.003	0.002	-0.003	0.009	0.632	0.025	0.023	-0.010	0.058	0.689
**MUSC**	0.01	0.003	0.002	-0.003	0.008	0.621	0.027	0.026	-0.010	0.062	0.692
**SKIN**	0.01	0.003	0.002	-0.002	0.007	0.623	0.022	0.021	-0.007	0.050	0.695
**BLOOD**	0.01	0.003	0.002	-0.003	0.007	0.620	0.030	0.029	-0.005	0.064	0.715
**ADIP**	0.05	0.019	0.002	-0.003	0.007	0.686	0.026	0.023	-0.008	0.061	0.692
**ARTERY**	0.05	0.004	0.003	-0.003	0.010	0.644	0.024	0.023	-0.009	0.057	0.688
**MUSC**	0.05	0.004	0.002	-0.003	0.008	0.610	0.026	0.025	-0.010	0.063	0.689
**SKIN**	0.05	0.003	0.002	-0.003	0.007	0.621	0.022	0.021	-0.010	0.053	0.688
**BLOOD**	0.05	0.003	0.002	-0.003	0.008	0.621	0.027	0.025	-0.005	0.060	0.719

Table S5: Cross-validation R^2 of ancestry-unaware models across MAF threshold. Related to Figure 2.

Tissue	MAF	Mean	Median	25% Quantile	75% Quantile
ADIP	0.00	0.005	0.003	8.56E-06	7.23E-03
ADIP	0.01	0.051	0.011	1.88E-03	5.21E-02
ADIP	0.05	0.049	0.010	1.83E-03	4.84E-02
ADIP	0.10	0.047	0.009	1.72E-03	4.51E-02
ARTERY	0.00	0.005	0.003	-4.12E-05	7.28E-03
ARTERY	0.01	0.056	0.011	2.17E-03	5.91E-02
ARTERY	0.05	0.054	0.011	1.97E-03	5.43E-02
ARTERY	0.10	0.052	0.010	1.86E-03	4.99E-02
BLOOD	0.00	0.005	0.002	2.20E-05	6.36E-03
BLOOD	0.01	0.047	0.009	1.79E-03	4.56E-02
BLOOD	0.05	0.045	0.009	1.57E-03	4.29E-02
BLOOD	0.10	0.043	0.008	1.44E-03	3.94E-02
MUSC	0.00	0.004	0.002	-5.80E-05	5.80E-03
MUSC	0.01	0.043	0.008	1.66E-03	4.17E-02
MUSC	0.05	0.041	0.008	1.48E-03	3.75E-02
MUSC	0.10	0.039	0.007	1.29E-03	3.53E-02
SKIN	0.00	0.005	0.002	-2.61E-05	6.79E-03
SKIN	0.01	0.051	0.010	1.79E-03	5.34E-02
SKIN	0.05	0.049	0.009	1.67E-03	4.97E-02
SKIN	0.10	0.047	0.009	1.71E-03	4.63E-02
